# Digital Engagement Strategy and Health Care Worker Mental Health: A Randomized Clinical Trial

**DOI:** 10.1001/jamanetworkopen.2024.10994

**Published:** 2024-05-24

**Authors:** Anish K. Agarwal, Lauren Southwick, Rachel E. Gonzales, Lisa M. Bellini, David A. Asch, Judy A. Shea, Nandita Mitra, Lin Yang, Michael Josephs, Michael Kopinksy, Rachel Kishton, Mohan Balachandran, Courtney Benjamin Wolk, Emily M. Becker-Haimes, Raina M. Merchant

**Affiliations:** 1 Department of Emergency Medicine, Perelman School of Medicine, Philadelphia, Pennsylvania; 2 Center for Digital Health, Penn Medicine, University of Pennsylvania, Philadelphia; 3 Center for Health Care Transformation and Innovation, Penn Medicine, University of Pennsylvania, Philadelphia; 4 Department of Medicine, Perelman School of Medicine, Philadelphia, Pennsylvania; 5 Department of Biostatistics, Epidemiology, and Informatics, Perelman School of Medicine, Philadelphia, Pennsylvania; 6 Department of Psychiatry, Perelman School of Medicine, Philadelphia, Pennsylvania

## Abstract

**Importance:**

The health care workforce continues to experience high rates of depression and anxiety. Finding ways to effectively support the mental health and well-being of health care workers is challenging.

**Objective:**

To test the effectiveness of remote, pushed digital assessments and engagement to improve depression and anxiety among health care workers compared with usual care.

**Design, Setting, and Participants:**

This was a 9-month randomized clinical trial with a 6-month intervention period. Participants were health care workers with self-reported daily access to a smartphone and at least 4 clinical hours per week. Participants were randomized to usual care or the intervention between January 2022 and March 2023. Data analyses were conducted between May and July 2023.

**Interventions:**

All participants completed baseline, 6-month, and 9-month mental health, well-being, and burnout assessments. The control group had open access to a web-based mental health platform. Participants in the intervention group received monthly text messaging about mental health, mental health assessments, and linkages to care.

**Main Outcomes and Measures:**

The primary outcomes were mean change in depression and anxiety scores at 6 months from baseline. Secondary outcomes include mean change in well-being, burnout, and self-reported workplace productivity.

**Results:**

In this study, 1275 participants were randomized (642 [50.4%] to the intervention group and 633 [49.6%] to control group). Participants had a mean (SD) age of 38.6 (10.9) years, 1063 participants (83.4%) were female, 320 (25.1%) self-identified as Black, and 793 (62.2%) self-identified as White. Across the groups, the mean difference in depression score was significantly different at 6 months (−0.96 [95% CI, −1.52 to −0.40]) and at 9 months (−1.14 [95% CI, −1.69 to −0.58]). The mean difference in anxiety score from baseline to 6 months was statistically significantly larger for those in the intervention group vs usual care (−0.71 [95% CI, −1.25 to −0.17]) and held true at 9 months (−1.06 [95% CI, −1.59 to −0.52]).

**Conclusions and Relevance:**

In a trial of health care workers, a proactive digital engagement strategy, including pushed text messaging, mobile mental health assessments, and connection to care, improved depression and anxiety over a 6-month period compared with simply making the same resources available for individuals to find and use.

**Trial Registration:**

ClinicalTrials.gov Identifier: NCT05028075

## Key Points

**Question:**

Can proactive digital engagement and connection to a well-being platform improve the mental health and well-being of health care workers?

**Findings:**

In this randomized clinical trial of 1275 health care workers, a proactive digital engagement strategy with a well-being platform modestly improved depression and anxiety during the 6-month intervention and persisted through 9 months.

**Meaning:**

These findings suggest that digital strategies to engage health care workers and connect them to resources when needed may reduce anxiety and depression.

## Teaser

This randomized clinical trial examines the effectiveness of using push notifications to engage health care workers and improve anxiety and depression.

## Introduction

The COVID-19 pandemic upended health care systems, exposing underlying crises in public health, medicine, and within the health care workforce itself.^1^ Rates of stress, burnout, and symptoms of anxiety and depression among clinician roles and nonclinical roles were high before the COVID-19 pandemic and worsened as a result of it.^2,3,4,5,6,7,8^ Adding to the complexity, these varying roles within the workforce have dynamic challenges and needs to help cope with the emotional and mental health strain of working within health care.

In response, health systems created online wellness platforms, which offer a variety of resources and content,^9,10^ for their clinical and nonclinical employees. These platforms can offer basic information, self-help resources, and more involved connection to expert mental health care, such as therapy.^11^ However, these online platforms, even if broadly publicized via typical channels such as email or meetings, still required individuals to recognize that they have an unmet mental health need and be aware of the resources available to them. Effectively, individuals must pull resources toward them in a setting when mental health needs are often unrecognized and, when they are, often put aside because of logistic challenges, concerns about stigma in health care,^12^ or any of the many reasons self-help is often avoided or delayed. An alternative strategy to overcome that inertia is to push resources to individuals in a proactive approach. A gap persists in understanding how to effectively support the mental health of a broad range of health care workers.

We conducted a clinical trial across a diverse group of health care workers comparing measures of depression and anxiety in those randomly allocated to a conventional pull vs a digital push strategy for mental health resources. The hypothesis of this study was that exposure to 6 months of a push strategy would make resources more readily available and lower depression and anxiety, which often co-occur.

## Methods

### Overview

Employees at an urban academic health system were recruited and randomized to usual care—the availability of mental health services—or the intervention, which consisted of usual care supplemented with pushed text messages with mental health assessments and linkage to those services. The intervention lasted for 6 months. The primary and secondary outcomes were measured at 6 and 9 months. The trial protocol (Supplement 1) was approved by the University of Pennsylvania’s institutional review board and followed the Consolidated Standards of Reporting Trials (CONSORT) reporting guideline.

The study used 2 platforms developed and designed at Penn Medicine, and both are available for use at other health care institutions.^9,13^ A well-being and mental health platform^9^ offered targeted content and individual and group support for the mental health and well-being of Penn Medicine workforce. Within the well-being and health platform, individuals could access videos, podcasts, and readings regarding well-being and mental health and assessments for anxiety, depression, stress, and well-being. The other platform^13^ was designed to facilitate remote engagement for clinical trials and clinical care, and participants completed baseline mental health, well-being, and self-reported work productivity assessments; this platform provided the ability to send messages pertaining to mental health reminders, links to resources and scheduling, and other study updates to participants in the intervention group.

### Participants

Eligible participants were aged 18 years or older, worked within the University Health System, were able to provide informed consent, were interested in participating in a 9-month study, worked at least 4 hours per week in a hospital or outpatient setting, and had regular and daily access to a smartphone. This was a health system–based research study and recruited across clinical roles beyond physicians and nurses. Participants were excluded if there was a condition that made it infeasible (eg, unable to provide informed consent), they did not speak English, did not have access to smartphone daily, or if they did not participate in at least 4 hours per week of patient care. Recruitment by email was conducted from January to May 2022. Emails were sent to a random sample of 10 000 potential participants from the estimated 40 000 employee pool within the University Health System. Eligible and consenting individuals were enrolled on a rolling basis and the final participants completed the 9-month follow up in March 2023. Given the unique and disproportionate strain on Black and female health care workers, these populations were oversampled in this study using specific requirement attention via established email listservs for a multiple of existing groups. This was accomplished by engaging diversity, equity, and inclusion leadership across the health system and specific email recruitment messaging to existing Black and female health care worker groups within the system. Participant race and ethnicity were self-reported from options including American Indian or Alaska Native, Asian, Black or African American, White, Native Hawaiian or Other Pacific Islander, and more than 1 race.

### Intervention Design

Participants enrolled in the usual care group were reminded that the mental health and well-being platform is a free resource available to all employees and were able to access it at any time. Participants enrolled in the intervention group received the following: (1) monthly automated text messages related to mental health and platform resources (eFigure 1 in Supplement 2) and (2) intermittent mental health assessments for depression (Patient Health Questionnaire [PHQ]–9) and anxiety (General Anxiety Disorder [GAD]–7). The monthly text messages provided popular well-being resources hosted on the platform. Upon completing the intermittent mental health assessments (eg, PHQ-9 and GAD-7), participants were connected to an appropriate platform resource according to their results (eFigure 2 in Supplement 2). If a participant was triaged to a mental health expert and prompted to schedule a session with a mental health professional, the automated text messaging system would send a follow-up message 2 days later asking whether the participant had booked a session or appointment.

### Baseline Assessments and Randomization

After completing baseline demographic and symptoms surveys, participants were randomized 1:1 in permuted random block sizes of 2, 4, and 6, stratified by race and sex to either usual care or intervention groups. Investigators were blinded to the group assignment, but participants were not.

### Outcome Measures

Depression and anxiety were assessed using the PHQ-9 and GAD-7, respectively. The PHQ-9 and GAD-7 are validated instruments and have been used in multiple studies and efficiently screen for symptoms of depression and anxiety.^14,15,16,17^ Well-being and burnout were measured using the Well-Being Index (WBI), a 9-question assessment validated for use in health care worker populations to assess burnout and the validated World Health Organization 5 Well-Being Index (WHO-5).^18,19,20^ Work productivity was measured using the Lam Employment Absence and Productivity Scale (LEAPS).^21^

The primary outcomes were depression (PHQ-9) and anxiety (GAD-7) change at 6 months from baseline. Secondary outcomes include 9-month change in PHQ-9 and GAD-7 and 6- and 9-month changes in well-being (WBI and WHO-5), and productivity (LEAPS) scores.

### Safety

A safety protocol was developed and approved by the institutional review board, and the study engaged a 3-member independent data and safety monitoring board. Participants reporting suicidal ideation via survey instruments or text messages were immediately contacted by the University Employee Assistance Program (EAP) for a safety assessment. Participants were contacted by EAP up to 3 times within 3 days. If any participant responded to a survey using words that were predetermined to be associated with self-harm (eg, death, kill, suicide, or hang), these individuals received an automated message directing them to seek emergency medical care if needed and providing suicide prevention resources (eFigure 3 in Supplement 2). All participants received text messaging sharing national suicide prevention information and how to connect to mental health resources upon the completion of the study.

### Statistical Analysis

We conducted an intent-to-treat (ITT) analysis. The study was powered to detect a 1-point difference in depression (PHQ-9) and anxiety (GAD-7) scales based on prior studies.^15,17,22^ We estimated a sample of 1275 participants would ensure 80% power, α = .05, to detect a 1-point difference and with the anticipation that there would be 20% attrition. Baseline demographic and clinical characteristics are reported as frequency and percentage for categorical variables and mean (SD) for continuous variables. We compared baseline characteristics between intervention and control groups using *t* tests for continuous variables, χ^2^ tests for categorical variables, and calculated standardized mean differences. We estimated and tested differences in means at 6 months and 9 months between groups using 2-sample *t* tests and generalized linear models to account for baseline measurements of PHQ-9 and GAD-7. The adjusted model is the generalized linear model adjusted by covariates including age, sex, race, ethnicity, marital status, and baseline anxiety or depression score. We used all available PHQ-9 and GAD-7 scores on eligible patients from randomization through the last observation. Data were imputed using multiple imputation with chained equations.^23^ Variables included in the imputation model were group assignment, age, sex, race, ethnicity, marital status, baseline anxiety or depression score, and for 9-month assessment the 6-month score was included. Analyses were completed using SAS software version 9.4 (SAS Institute) and conducted between May and July 2023. Statistical significance was set at *P* < .05, and tests were 2-sided.

## Results

**Figure 1.**
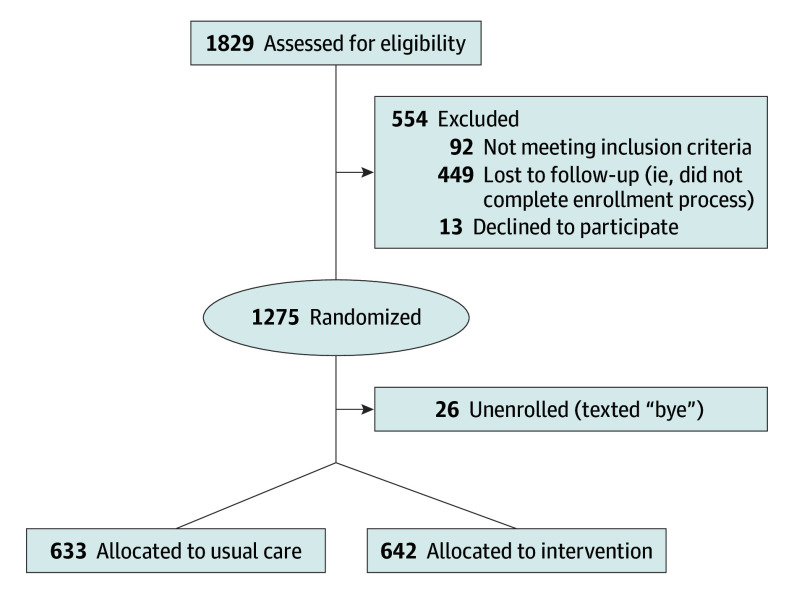
CONSORT Diagram

In this study, 10 000 health care workers were randomly invited to participate in the study by email, and using a rolling enrollment process, 1854 health care workers were assessed for eligibility and 1275 (68.7%) were randomized (642 to the intervention group and 633 to the usual care group) (Figure 1). Participants had a mean (SD) age of 38.6 (10.9) years, 1063 participants (83.4%) were women, 320 (25.1%) self-identified as Black, and 793 (62.2%) self-identified as White. Participant characteristics were balanced across the groups with no significant differences in age, sex, race, ethnicity, marital status, or clinical role (Table 1). Across both groups, there were 416 nurses (33%), 174 physicians (14%), 151 technicians (12%), and 122 registrars (10%) (eTable 1 in Supplement 2). Mean (SD) baseline GAD-7 score was 5.79 (4.77) in the control group and 6.03 (4.88) in the intervention group. Mean (SD) baseline PHQ-9 score was 5.71 (4.74) in the control group and 5.92 (5.19) in the intervention group. Table 2 displays the proportion of scores and means by severity between groups.

**Table 1.** Participant Demographics

Characteristic	Participants, No. (%)		
	Control (n = 633)	Intervention (n = 642)	Total (N = 1275)
Age, mean (SD), y	38.6 (11.1)	38.6 (10.6)	38.6 (10.9)
Age group, y			
18-35	305 (48.2)	296 (46.1)	601 (47.1)
36-50	219 (34.6)	249 (38.8)	468 (36.7)
51-64	100 (15.8)	86 (13.4)	186 (14.6)
≥65	9 (1.4)	11 (1.7)	20 (1.6)
Sex			
Female	529 (83.6)	534 (83.2)	1063 (83.4)
Male	104 (16.4)	108 (16.8)	212 (16.6)
Race and ethnicity			
Asian	54 (8.5)	55 (8.6)	109 (8.5)
Black	160 (25.3)	160 (24.9)	320 (25.1)
Hispanic	38 (6.0)	39 (6.1)	77 (6.0)
White	394 (62.2)	399 (62.1)	793 (62.2)
Other^a^	25 (3.9)	28 (4.4)	53 (4.2)
Married or with partner	352 (55.6)	367(57.2)	719 (56.4)
Shiftwork	283 (44.7)	267 (41.6)	550 (43.1)
Manager	137 (21.6)	153 (23.8)	290 (22.7)
Profession role			
Physician	85 (13.4)	89 (13.9)	174 (13.6)
Nurse	210 (33.2)	206 (32.1)	416 (32.6)
Other^b^	338 (53.4)	347 (54.0)	685 (53.7)
Baseline survey score, mean (SD)			
Anxiety (GAD-7)	5.79 (4.77)	6.03 (4.88)	5.91 (4.83)
Depression (PHQ-9)	5.71 (4.74)	5.92 (5.19)	5.81 (4.97)
Burnout (WBI)	2.70 (2.33)	2.72 (2.23)	2.71 (2.28)
Well-being (WHO-5)	13.13 (5.33)	13.10 (5.26)	13.12 (5.29)
Work productivity (LEAPS)	4.43 (4.00)	4.51 (4.28)	4.47 (4.14)

Abbreviations: GAD-7, General Anxiety Disorder; LEAPS, Lam Employment Absence and Productivity Scale; PHQ-9, Patient Health Questionnaire; WBI, Well-Being Index; WHO-5, World Health Organization 5 Well-Being Index.

^a^ Other race includes American Indian or Alaska Native, Native Hawaiian or Other Pacific Islander, and more than 1 race.

^b^ See eAppendix in Supplement 2 for breakdown.

**Table 2.** Baseline Anxiety and Depression Assessments

Assessment	Participants, No. (%)			*P* value
	Control (n = 633)	Intervention (n = 642)	Total (N = 1275)	
Anxiety				
Minimal or mild	513 (81.0)	512 (79.8)	1025 (80.4)	.80
Moderate	78 (12.3)	82 (12.8)	160 (12.5)	
Severe	42 (6.6)	48 (7.5)	90 (7.1)	
Anxiety score, mean (SD)				
Minimal or mild	3.94 (2.77)	3.99 (2.59)	3.96 (2.68)	.78
Moderate	11.69 (1.39)	12.04 (1.44)	11.87 (1.42)	.13
Severe	17.43 (1.71)	17.58 (1.97)	17.51 (1.84)	.69
Depression				
Minimal or mild	518 (81.8)	506 (78.8)	1024 (80.3)	.21
Moderate	77 (12.2)	87 (13.6)	164 (12.9)	
Moderately severe	30 (4.7)	31 (4.8)	61 (4.8)	
Severe	8 (1.3)	18 (2.8)	26 (2.0)	
Depression score, mean (SD)				
Minimal or mild	3.97 (2.83)	3.72 (2.66)	3.85 (2.75)	.16
Moderate	11.51 (1.32)	11.59 (1.40)	11.55 (1.36)	.71
Moderately severe	16.60 (1.45)	16.42 (1.36)	16.51 (1.40)	.62
Severe	21.88 (1.55)	22.17 (1.79)	22.08 (1.70)	.69

### Anxiety and Depression

Most participants had minimal or mild anxiety at baseline (1052 participants [80.4%]). The mean 6-month change in GAD-7 score was −0.28 in the control group and −0.99 in the intervention group (difference, −0.71 [95% CI, −1.25 to −0.17]). The mean (SD) GAD-7 score for the control group was 5.79 (4.77) at baseline and decreased to 5.54 (4.81) at 6 months, which was not significant. Of those in the control group completing the GAD-7 at 9 months (492 participants [77.7%]), the baseline mean (SD) score was 5.60 (4.61) and decreased significantly to 4.85 (4.47) (difference, −0.74; 95% CI, −1.13 to −0.36). The mean (SD) GAD-7 score in the intervention group was 5.99 (4.85) at baseline, which significantly decreased to 5.00 (4.90) at 6 months (difference, −0.99 [95% CI, −1.41 to −0.57]); of those who completed the assessment at 9 months (467 participants [72.7%]), the mean (SD) score significantly decreased to 4.18 (4.51) (difference, −1.80; 95% CI, −2.17 to −1.43) (Table 3). These findings were consistent using imputed data and the adjusted model (eTable 2 in Supplement 2).

**Table 3.** Unadjusted Change in Mean Anxiety and Depression Between Intervention and Control Groups at 6 Months and 9 Months

Measure	Score, mean (SD)			Difference (95% CI)	*P* value
	Control	Intervention	Total		
Anxiety					
Participants, No. (%)	633 (49.6)	642 (50.4)	1275 (100.0)	NA	.37
GAD-7	5.79 (4.77)	6.03 (4.88)	5.91 (4.83)	0.24 (−0.29 to 0.77)	
6-mo Follow-up					
Responding participants, No. (%)	509 (52.4)	462 (47.6)	971 (100.0)	NA	NA
Baseline GAD-7	5.82 (4.75)	5.99 (4.85)	5.90 (4.80)	0.17 (−0.43 to 0.78)	.57
6-mo GAD-7	5.54 (4.81)	5.00 (4.90)	5.28 (4.86)	−0.54 (−1.15 to 0.07)	.08
6-mo Difference (95% CI)	−0.28 (−0.63 to 0.07)	−0.99 (−1.41 to −0.57)	−0.62 (−0.89 to −0.35)	−0.71 (−1.25 to-0.17)	<.001
9-mo Follow-up					
Responding participants, No.	492 (51.3)	467 (48.7)	959 (100.0)	NA	
Baseline GAD-7	5.60 (4.61)	5.98 (4.84)	5.78 (4.72)	0.38 (−0.22 to 0.98)	.21
9-mo GAD-7	4.85 (4.47)	4.18 (4.51)	4.53 (4.50)	−0.67 (−1.24 to −0.10)	.02
9-mo Difference (95% CI)	−0.74 (−1.13 to −0.36)	−1.80 (−2.17 to −1.43)	−1.26 (−1.53 to −0.99)	−1.06 (−1.59 to −0.52)	<.001
Depression					
Participants, No.	633 (49.6)	642 (50.4)	1275 (100.0)	NA	.45
PHQ-9	5.71 (4.74)	5.92 (5.19)	5.81 (4.97)	0.21 (−0.34 to 0.76)	
6-mo Follow-up					
Responding participants, No.	508 (52.4)	462 (47.6)	970 (100.0)	NA	NA
Baseline PHQ-9	5.59 (4.70)	5.91 (5.26)	5.75 (4.97)	0.32 (−0.31 to 0.95)	.32
6-mo PHQ-9	5.29 (4.93)	4.65 (4.73)	4.99 (4.85)	−0.64 (−1.25 to −0.03)	.04
6-mo Difference (95% CI)	−0.30 (−0.67 to 0.06)	−1.26 (−1.69 to −0.83)	−0.76 (−1.04 to −0.48)	−0.96 (−1.52 to −0.40)	<.001
9-mo Follow-up					
Responding participants, No.	492 (51.3)	467 (48.7)	959 (100.0)	NA	NA
Baseline PHQ-9 (SD)	5.51 (4.64)	5.84 (5.20)	5.67 (4.92)	0.33 (−0.30 to 0.95)	.31
9-mo PHQ-9 (SD)	4.87 (4.87)	4.06 (4.74)	4.48 (4.82)	−0.81 (−1.42 to −0.20)	.009
9-mo Difference (95% CI)	−0.64 (−1.03 to −0.25)	−1.78 (−2.17 to −1.38)	−1.19 (−1.47 to −0.91)	−1.14 (−1.69 to −0.58)	<.001

Abbreviations: GAD-7, General Anxiety Disorder; NA, not applicable; PHQ-9, Patient Health Questionnaire.

**Figure 2.**
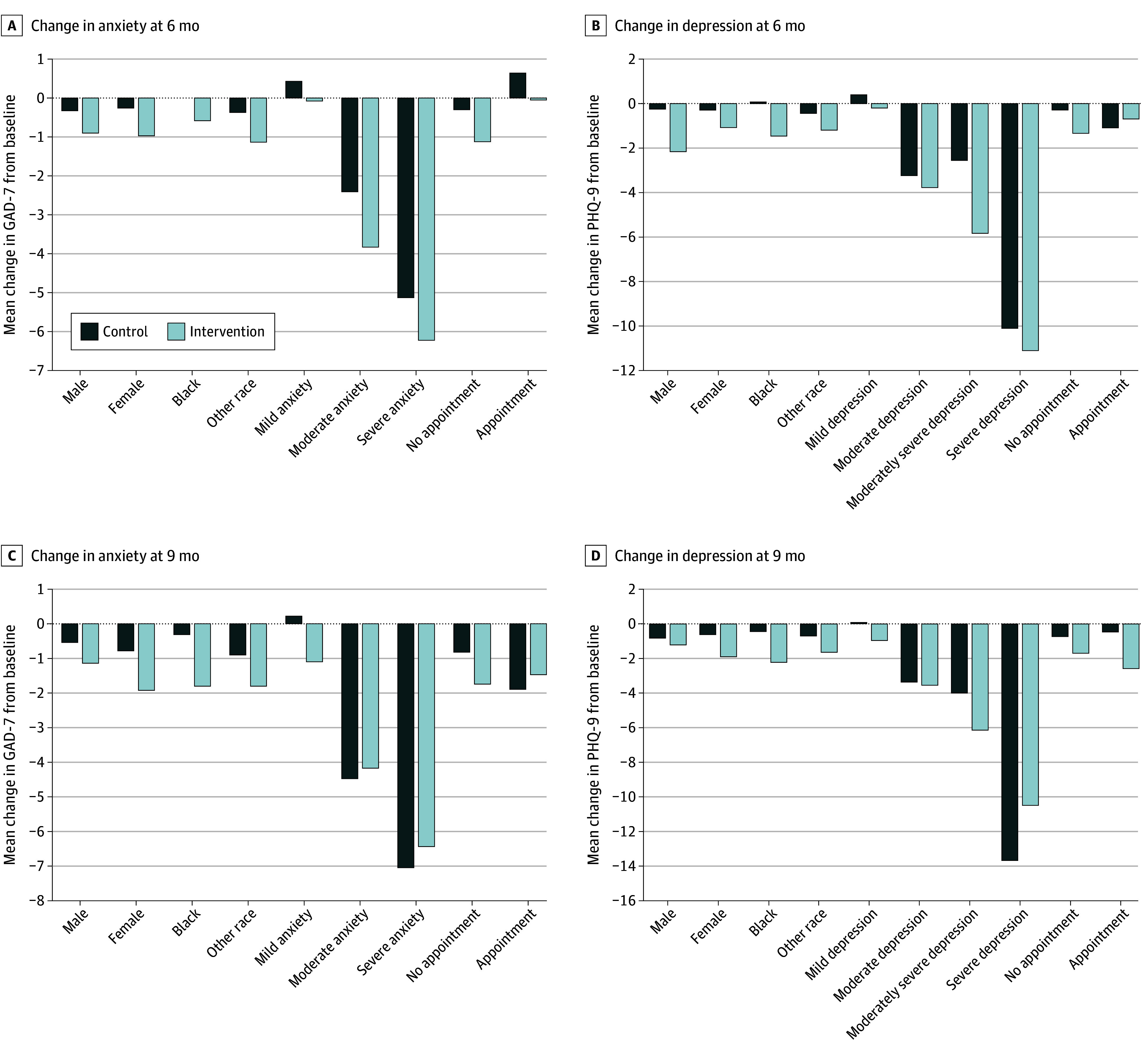
Change in Anxiety and Depression at 6 and 9 Months Change in mean anxiety and depression between intervention and control groups at 6 and 9 months.

In subgroup analyses, significant decreases in anxiety were seen at the 6- and 9-month periods for female participants, Black participants, and those with minimal or mild baseline anxiety scores (Figure 2). Most participants had minimal or mild depression scores (1024 participants [80.3%]). The mean 6-month change in PHQ-9 score was −0.30 in the control group and −1.26 in the intervention group (difference, −0.96; 95% CI, −1.52 to −0.40). The mean (SD) PHQ-9 score for the control group was 5.59 (4.70) at baseline and 5.29 (4.93) at 6 months, for a difference of −0.30 (95% CI, −0.67 to 0.06). Of those completing the 9-month assessment (492 participants [77.7%]), the mean (SD) score was 5.51 (4.64) at baseline and significantly decreased to 4.87 (4.87), for a mean difference of −0.64 (95% CI, −1.03 to −0.25). In the intervention group, the mean (SD) PHQ-9 score was 5.91 (5.26) at baseline and significantly decreased to 4.65 (4.63), a mean difference of −1.26 (95% CI, −1.69 to −0.83). The mean (SD) PHQ-9 score was 5.84 (5.20) at baseline and significantly decreased to 4.06 (4.74) at the 9-month assessment, a difference of −1.78 (95% CI, −2.17 to −1.38). These results were consistent using imputed data and in the adjusted model (eTable 2 in Supplement 2). To account for multiple measurements over time we also completed a generalized estimating equations for repeated measures analysis (eTable 6 in Supplement 2). Figure 1 displays scores over time for the intervention and control groups. Across groups, the mean difference was significantly different at 6 months (−0.96 [95% CI, −1.52 to −0.40]) and at 9 months (−1.14 [95% CI, −1.69 to −0.58]). At the 2 follow-up periods, significant decreases in depression were seen among female participants, male participants at 6 months only, and those with minimal or mild depression at 6 months (Figure 1).

### Well-Being and Burnout

The mean (SD) WBI score in the control group was 2.70 (2.32) at baseline and decreased significantly to 2.24 (2.30) at 6 months, with higher scores indicating more distress. In the intervention, the mean (SD) score was 2.64 (2.22) at baseline and significantly decreased to 2.05 (2.34) at 6 months. There was no significant difference between groups at 6 months (eTable 3 in Supplement 2).

The WHO-5 mean (SD) score in the control group was 13.13 (5.33) at baseline and increased to 13.88 (5.23) at 6 months, with higher scores indicating improvement. The mean (SD) WHO-5 in the intervention group was 13.10 (5.26) at baseline and increased to 14.15 (5.43) at 9 months. All of these findings were confirmed in imputed and adjusted analyses. There was no statistically significant difference between the control and intervention groups at 6 months (eTable 4 in Supplement 2).

### Work Productivity

The control group had a mean (SD) LEAPS score of 4.43 (4.00) at baseline, and this was significantly higher at 5.25 (4.08) at 6 months, with higher scores indicating more severe work impairment. Of those completing the 9-month assessment, the mean (SD) baseline score was 4.40 (3.94) and significantly increased to 5.15 (4.30). The intervention group mean (SD) score was 4.51 (4.28) at baseline, which increased significantly to 4.94 (4.34) at 6 months. At 9 months, the mean (SD) score was 4.17 (3.93), which decreased from 4.45 (4.28) for those completing baseline and 9-month assessments (eTable 5 in Supplement 2). Across groups, the mean difference between control and intervention groups showed a mean reduction of −1.02 (95% CI, −1.58 to −0.47) at 9 months.

### Suicidal Ideation

The final question of the PHQ-9 assessment evaluates for suicidal ideation. Throughout the study, 164 participants (12.8%) expressed suicidal ideation across 212 assessments (5.2%). The University EAP evaluated and triaged 127 participants (59.9%) to an appropriate level of care. Of these 127 participants, 85 (40.0%) did not respond to 1 of 3 separate phone calls for follow-up by employee assistance and received safety materials again. Of these 85 participants, 57 (67.0%) responded to a study message or completed an additional assessment after screening positive for suicidal ideation, 18 (21.1%) interacted with study compensation gift cards, and 8 (9.4%) did not engage in any further study activities. Two individuals unenrolled from the study; 1 was assessed and the other was not assessed but responded to a final research study message (eFigure 4 in Supplement 2). No text messaging safety trigger word events occurred.

## Discussion

In this randomized clinical trial, we found that a digital engagement strategy that pushed intermittent well-being and mental health messaging, connection to care, and remote assessments was associated with modest improvements in depression and anxiety compared with usual care among health care workers. The magnitude of effect associated with depression and anxiety was small but was sustained 3 months after the intervention. There were no significant changes in well-being or burnout measures at 6 months.

These findings build upon the expanding literature of the well-being and mental health of the health care workforce. Within the larger context of health workforce depletion, rising levels of burnout, and persistent stigma in acknowledging and addressing mental health symptoms among health care workers, new strategies are needed to support this at-risk group. As health systems search for ways to promote engagement and belonging, some have implemented online platforms to centralize and make well-being and mental health resources available.^9,24,25^ Popularizing the actual use of these platforms can be challenging. This trial tested strategies that were designed to proactively engage the workforce using simple text message assessments, links to resources, and mobile mental health assessments, triaging individuals to expert care on the basis of their responses. Prior research^12,26^ has aimed to build online resource hubs for the workforce; however, barriers persist in helping individuals complete assessments and react to them in real time. The current findings suggest that an approach that pushes content can be supportive for enabling individuals to connect to mental health resources and care when needed and may promote engagement in a way that is more accessible. This personalized approach builds on research to tailor evidence-based care in a manner that helps unique populations.^12,27^

In this study, we also highlighted a novel approach of proactively and longitudinally assessing symptoms of mental health distress and well-being across the workforce. The study sent out email recruitment to a random selection of 10 000 individuals across clinical roles to represent a pragmatic cohort beyond physicians and nurses (eg, technicians, registrars, and others). In this study, enrolled participants generally reflected the makeup of the local workforce, including the majority being females and nurses. Historically, health systems have relied on annual or semiannual large-scale surveys to assess clinician burnout. This study used intermittent remote mental health assessments to evaluate the trajectories of mental health symptoms and well-being. Here, we do not demonstrate a clinically significant change in well-being scores and worsening productivity scores, although some remain statistically significant. These findings shed light on the need for organizational and operational change to empower the workforce with structural support. Although the intervention and control groups did not change in their well-being outcomes, we did begin to see evidence of the feasibility of intermittent, low friction, and rapid assessment of a large workforce to understand and better characterize variations over time. Future studies testing tailored and targeted interventions toward specific clinical roles may reveal larger and longer effects associated with well-being and burnout.

Finally, we demonstrated that the persistent mild-to-moderate amount of anxiety and depression across the health care workforce was higher than those reported within the general US population.^28,29^ The mental health resources made available to participants in this study existed since the early stages of the COVID-19 pandemic and mirror that of many other large health systems, including a centralized resource for information, group therapy, or 1-on-1 mental health counseling. Notably, the intervention did not change the resources or experts available to individuals; however, it did bring this content closer to the end user. Indeed, we identified improvements across control and intervention groups in anxiety and depression at 9 months, which signals a trend toward global improvement, though the effect was larger in the intervention group. Addressing the persistent mental health symptoms is critical within the health care infrastructure. The strategy and findings reveal signals that these symptoms can be identified early and acted upon at scale with reductions in anxiety and depression. Future studies will need to explore how to reach high-risk individuals before the development of serious mental health disorders and burnout. In this sample, 1 of 8 health care workers expressed suicidal thoughts. This is higher than other published studies during the initial phases of the COVID-19 pandemic^30^ and poses a real threat to the health care workforce. Thus, a strategy that helps identify and support these individuals is critical to the success of the industry and for patient care.

### Strengths and Limitations

This study has several strengths. The intervention provided a remote, simple, and scalable strategy to engage and support the mental health of the health workforce. The sample was unique in that it extended beyond physicians and nurses, on whom much previous research has focused, and included the broader workforce (eg, pharmacists and social workers). The program did not change the mental health or well-being resources available to the study population, but rather the ways in which the individuals were reminded and navigated to support resources. The study builds on literature studying the mechanisms to lower the barriers to mental health care and highlights high rates of suicidal ideation in medicine (1 in 8 of participants).

The study also has limitations. First, participants were from a single, large, urban health system and needed daily access to a smartphone, which may limit generalizability, especially within the context of the known racial and ethnic digital divide. However, this divide may be smaller among health care workers who are employed, and smartphone ownership continues to expand in the US. Second, the recruitment used a randomly selected group of 10 000 health care workers across a large system that employs approximately 40 000 individuals. Interested participants were screened for eligibility and enrolled. Therefore, selection and nonresponder bias is present. This is especially true for mental health study in an environment where documented stigma exists in seeking mental health support. Third, to promote participant privacy, this study did not integrate mental health records or treatments received but rather gathered self-reported data from participants. Future studies will need to explore diagnoses by mental health professionals and longer term follow-up regarding mental health outcomes. Fourth, the sample was largely female, which may limit generalizability but may also reflect the makeup of many health system employee populations. Fifth, there was attrition in those completing the 6-month and 9-month follow-up surveys (approximately 25%); however, we found no significant differences in the primary outcomes using our imputed analysis.

## Conclusion

In this randomized clinical trial of health care workers, a proactive digital engagement strategy, including pushed text messaging, mobile mental health assessments, and connection to care, improved depression and anxiety during a 6-month period compared with simply making the same resources available. These findings were sustained at the 9-month follow-up. Future interventions could test tailored support for specific mental health symptoms, suicidal ideation, and well-being to lead to personalized support for the health workforce.

## Notes

Supplement 1.
Trial Protocol

Supplement 2. **eFigure 1.** Monthly Automated Text Messages Associated With Mental Health and Penn Cobalt Resources And Mental Health Assessments (e.g., PHQ-9 and GAD-7) Appointment Triage Text Messages and Roles **eFigure 2.** Mental Health Assessments (e.g., PHQ-9 and GAD-7) Appointment Triage Messaging **eFigure 3.** Safety and Suicidality **eFigure 4.** Safety and Suicidality Findings **eTable 1.** GLM Model on Complete Cases: 6-Month GAD-7 **eTable 2.** Anxiety and Depression Comparison With Imputed Data **eTable 3.** Well-Being Index Comparison With Observed Data **eTable 4.** WHO-5 Comparison With Observed Data **eTable 5.** Leaps Comparison With Observed Data **eTable 6.** GEE With AR(1)

Supplement 3.
Data Sharing Statement
