# Supplementary material for: Digital Engagement Strategy and Health Care Worker Mental Health: A Randomized Clinical Trial
Source: JAMA Netw Open. 2024 May 24;7(5):e2410994. doi: 10.1001/jamanetworkopen.2024.10994 (PMC11127125; doi:10.1001/jamanetworkopen.2024.10994)
Supplement: Supplement 1. — Trial Protocol [file jamanetwopen-e2410994-s001.pdf]

Supplement

Trial Protocol and Statistical Analysis Plan

Effect of a Digital Engagement Strategy on Healthcare Worker Mental Health and Well-being: A Randomized Controlled Trial

Anish K. Agarwal MD, MPH, MS; Lauren Southwick MPH; Rachel Gonzales MPH; Lisa Bellini MD; David A Asch MD, MBA; Judy Shea PhD; Nandita Mitra PhD; Lin Yang MS; Michael Josephs BS; Michael Kopinksy BS; Mohan Balachandran MA, MS; Courtney Wolk MD; Emily Becker-Haimes PhD; Raina M Merchant MD, MSHP

Page

Original protocol 3
Final protocol 20
Summary of protocol changes 44
Original statistical analysis plan 53
Final statistical analysis plan 54
Summary of statistical analysis plan changes 55

Original Study Protocol

Table of Contents

1. **Abstract**……3
2. **Background**……3
3. **Overall Objectives**……5
3.1. Primary Aims……5
3.2. Secondary Aims……5
4. **Primary Outcome Variable**……5
5. **Secondary Outcome Variable(s)**……5
6. **Study Design**……5
6.1. Phase……5
6.2. Design……6
6.3. Study Duration……7
6.4. Facilities……7
6.5. Key Inclusion Criteria……7
6.6. Key Exclusion Criteria……8
7. **Subject Recruitment**……8
7.1. Target Population……8
7.2. Subjects at Penn……8
7.3. Accrual……8
7.4. Patient Subject Recruitment……8
7.5. Subject Compensation……9
8. **Study Procedures**……9
8.1. Consent Process……9
8.1.1. Waiver of Alteration of Informed Consent……10
8.1.2. Minimal Risk……10
8.1.3. Impact on Subject Rights and Welfare……10
8.1.4. Waiver Essential to Research……11
8.1.5. Written Statement of Research……11
8.2. Procedures……11
9. **Analysis Plan**……11
10. **Subject Confidentiality**……12
10.1. Subject Privacy……13
10.2. Data Disclosure……13
10.3. Data confidentiality……13
11. **Consent Process Overview**……**14**
11.1. Potential Study Risks……14
11.2. Potential Study Benefits……14
11.3. Alternatives to Participation……15
11.4. Data Safety and Monitoring……15

1. Abstract

Early in the pandemic, our team developed and implemented Penn Cobalt across the University of Pennsylvania Health System which spans six acute-care hospitals and employs over 43,000 individuals. Cobalt is a web-based platform that curates mental health and wellness content and provides connection to group and individual support. Using validated mental health assessments, Cobalt triages users to the right level and type of support across three categories: In the studio synchronous group sessions, On your time asynchronous content, and 1:1 support. The 1:1 support represents a stepped care model of mental health services and includes peer support, resilience coaches offering psychological first aid, psychotherapists, and psychiatrists. Cobalt's embedded scheduling and telehealth capabilities also provide HIPAA-compliant mental health care in a convenient, patient-centered model. Cobalt content continuously adapts and evolves through crowdsourced feedback to encompass locally defined and sensitive resources (e.g. addressing racial trauma). Over the first 7 months, Cobalt has had over 140,000-page views and 18,300 unique users engaging with its content and support. The platform identified 111 HCWs reporting thoughts of self-harm and connected those individuals with a mental health provider for support and evaluation within 24 hours.

1. Background

Epidemics are often associated with significant mental health consequences to society. Large-scale disasters, whether traumatic (e.g., the World Trade Center attacks or mass shootings), natural (e.g., hurricanes), or environmental (e.g., Deepwater Horizon oil spill), are often accompanied by increases in depression, posttraumatic stress disorder (PTSD), substance use disorder, and a broad range of other mental health and behavioral disorders, domestic violence, and child abuse. For example, prior work showed that 5% of the population affected by Hurricane Ike in 2008 met the criteria for major depressive disorder in the month after the hurricane; 1 out of 10 adults in New York City showed signs of the disorder in the month following the 9/11 attacks. And almost 25% of New Yorkers reported increased alcohol use after the tragic attacks. Communities affected by the Deepwater Horizon oil spill showed signs of clinically significant depression and anxiety. The SARS epidemic was also associated with increases in PTSD, stress, and psychological distress in patients and clinicians. For such events, the impact on mental health can occur in the immediate aftermath and then persist over long time periods. The rates of stress and burnout were high in health care workers prior to the pandemic and those rates have persisted or increased. In March of 2020, COVID-19 emerged as a global pandemic resulting in acute stressors directly impacting an already vulnerable and strained health care system. Healthcare workers (HCWs) are facing unique challenges during the COVID-19 pandemic related to rapid shifts in care, strains on availability of personal protective equipment, moral injury, and concerns about risks of infection. The challenges confronting HCWs continue outside of work including childcare responsibilities, career trajectory and concern for employment status with dynamically shifting practice models and patient volumes. The mental health burdens of frontline HCWs began to emerge in early retrospective studies from China and Italy; with early reports of increases in clinician suicide due to the pandemic. HCWs are predicted to face repeated acute triggering events throughout the long period of disillusionment we now find ourselves in as we slowly inch towards reconstruction and recovery with the COVID-19 pandemic. Additional HCW stress is associated with the real or perceived risk of contracting COVID-19 or spreading it to loved ones. A 2020 MMWR report on occupation type and job setting of HCWs with COVID-19 showed that the relative percentage of cases was high in nurses (1742, 29.5%), environmental services (EVS) (330, 5.6%), physicians (190,3.2), and respiratory therapists (44, 0.7%). COVID-19 has also affected HCWs across demographics with higher rates of death in underrepresented minorities. Among HCW with COVID-19 the median age was 41 years and 79% were females. For the 69,678 (69%) of cases with available data on race and ethnicity the distribution was Whites (47%), Blacks (26%), Hispanics (12%) Asians (9%). Compared with COVID-19 survivors, non-

survivors were more likely to be older (median age 62), male (38% vs. 22%), Asian (20% vs. 9%), or Black (32% versus 25%). The increased exposure to COVID-19 may also limit physical contact of HCW with usual support networks and systems (e.g. family or faith-based groups) or lead to increased stress because of home based caregiving for elderly or immunocompromised family members. HCWs identifying as Black, indigenous, or of color are a vulnerable population. Prior reports support that black adults are approximately 10% more likely to report psychological distress when compared with white adults. Data from the Health and Human Services Office of Minority Health also shows that black adults are more likely than white adults to report hopelessness, sadness, and feeling like everything is an effort. There are significant barriers to accessing mental health care for black adults and it is estimated that only one in three black adults who need mental health care receive it. Many reasons contribute to these disparities including: costs of care, stigma associated with care, provider bias, lack of knowledge regarding available mental health therapies, and the lack of minority mental health professionals. For those who do receive care, the care is often of lower quality and not culturally aligned. Black HCWs have historically been subject to racism, discrimination, and inequity--and the national events centered around racial injustice coupled with COVID-19 have likely exacerbated mental health and well-being in measurable and sustained ways. Minority HCWs were already strained, often working within settings where they themselves are subject to microaggressions, overt acts of racism, structural racism and are direct observers of racial inequities in patient care leading to significant disparities in morbidity and mortality. Racial minorities are also disproportionately in jobs with high exposure risk including grocery stores, public transportation, factories, and health care facilities. A meta-analysis of 293 studies identified that racism significantly contributed to worse mental and physical health. Based on findings from more than 60 structured interviews with Black HCWs, the noted sociologist Adia Wingfield summarized her findings and noted: being a Black health care worker comes with specific difficulties that can easily go unnoticed. In a pandemic where Black populations are among the hardest hit, these difficulties are likely being magnified exponentially. Health care systems are undoubtedly taxed, but in the interest of their workers, they should consider ways they can support Black health care providers to offset the kinds of burnout and stresses research indicates they are likely experiencing right now. In this national emergency, health care systems may need to think past providing health care just for patients and consider the health of their workers, perhaps through counseling and support groups, heeding employees suggestions for how systems can be improved. The social and economic consequences of COVID-19 are predicted to affect women differently than men. Prior reports support that women have been more likely to lose their jobs or leave them during the pandemic due to a need to provide child and elder care as well as to support home schooling. While this unpaid labor has increased, paid work opportunities have also decreased for women. Increases in sexual and physical violence toward women during lockdowns have also been reported. The United Nations has identified increased vulnerability of female frontline workers and the need for targeted programs to specifically address their unique mental health needs due to a multitude of stressors. Prior reports have also focused on female frontline providers and physician researchers and threats to their professional careers, particularly academic, advancement due to barriers in work productivity as a result of COVID-19. The psychological impact of the delay in academic career progression (e.g. fewer grant submissions, fewer peer-reviewed publications, fewer leadership opportunities) could be pronounced and long standing. Considering how the pandemic has led to considerable strain on women and the unique impact on women in health care, this proposal specifically focuses on evaluating if Cobalt+ will have a greater impact on women and can serve as a platform for addressing inequities in mental health care for this vulnerable population.
COVID-19 has increased the use and application of digital technologies in health care including text messaging, mobile surveys, and telemedicine. Evolving and rapidly adopted approaches in mobile engagement through digital technology create scalable opportunities to assess individuals needs in real-time and can be used to rapidly deploy tailored well-being and mental health interventions. This

approach also offers a strategy to reveal the central tendencies, distributions, and associations which, in population-based studies, can immediately inform a health systems approach toward maintaining a healthy workforce and lowering barriers to mental health care.

2. Overall Objectives

Through a RCT, evaluate the effectiveness of Cobalt+ on HCW depression/anxiety (primary outcome) compared with Cobalt (usual care).

2.1. Primary Aims

Aim 1: Through a RCT, evaluate the effectiveness of Cobalt+ on HCW depression/anxiety (primary outcome) compared with Cobalt (usual care).

Aim 2: To better understand perceptions of access to mental health care and the effectiveness of Cobalt compared with Cobalt+ among HCWs through semi-structured qualitative interviews.

2.2. Secondary Aims

Secondary outcomes will include well-being, satisfaction with access to care, and measures of work productivity. HTE will be explored for race and gender.

3. Primary Outcome Variable

Measures were selected based on clinical relevance, validity in the HCW population, and meaningfulness to the target population. To facilitate data sharing, measures below were also identified to align with the NIMH data archive when possible Depression and Anxiety: The primary outcome will be depression and anxiety as measured by the Patient Health Questionnaire (PHQ-9) and General Anxiety Disorder (GAD-7) respectively. PHQ-9 and GAD-7 have been validated and used in multiple studies and provide easy, simple scales to quickly assess for depression and anxiety. PHQ9 is a 9-item score which ranges from 0 (least depressed) to 27 (most depressed. GAD-7 is a 7- item scale which assess the frequency of anxiety symptoms over the past two weeks on a 4-point Likert-scale ranging from 0 (never) to 3 (nearly every day). The total score of GAD-7 ranged from 0 to 21, with increasing scores indicating more severe functional impairments as a result of anxiety.

4. Secondary Outcome Variable(s)

Well-being index: The Well-being index (WBI) is a nine-question survey validated for use in HCW populations and considered important to health systems in managing the well-being of their workforce. Work productivity: To evaluate work productivity, we will use the World Health Organization Health and Work Performance Questionnaire (HPQ) short form. The HPQ is a validated self-report instrument designed to estimate the workplace costs of health problems with regard to reduced job performance, absence due to sickness, and work-related injuries and accidents. We will administer the short form which is an 11-question survey focused on absenteeism and presenteeism. Satisfaction with care: Client Satisfaction with Care (CSQ)-8 is a brief 8-item unidimensional well-established scale for assessing patients satisfaction with overall outpatient health care. It has been shown to be reliable and valid in assessing care across a range of settings.

5. Study Design

5.1. Phase

Not applicable

5.2. Design

We will conduct a prospective two-armed randomized control trial to test the effect of tailored mental health support on self-reported HCW mental health (anxiety and depression) during the response and recovery phases of the COVID-19 pandemic. The control group will have access to Cobalt which is usual care for all Penn Medicine employees. The Cobalt platform is web-based and is accessible from any device or desktop. Cobalt uses contextual surveys and evidence-based assessments to triage individual users to curated content and appropriate group or individual support. The current, and usual care, model is a digital version of what exists at other institutions where resources are available for staff to access wellness and mental health services. The usual care Cobalt model requires staff to personally initiate multiple steps: 1) identify a personal need for support, 2) know where to seek support and care, 3) schedule an appointment, and 4) attend the appointment. In this context, the individual has to pull the resources they need and there may be several barriers to completing each step. This is particularly relevant as mental health conditions can compromise insight, motivation, and decision making, thereby making self-directed engagement in care (pull) more challenging. The study will be single-blinded and the study biostatistician will determine when to unblind the study. Given that the study compares the same process delivered as either a pull (usual care = Cobalt) or a push (intervention = Cobalt +) we believe risk is low. Nevertheless, we will engage a 3-member independent Data and Safety Monitoring Board to oversee the trial. In accordance with conventional practices, we will not identify members in advance so as not to disqualify potential proposal reviewers. To assess factors that may contribute toward accessing and using mental health resources between Cobalt versus Cobalt+, we will conduct semi-structured interviews (telephone or virtual video conference) with HCWs enrolled in Aim 1. Standardized interview guides will be created using the consolidated framework for implementation research (see Appendix) to ensure uniformity in how and what questions are asked of each group. Some questions within the guides will be the same between those in the traditional care arm (Cobalt) versus intervention (Cobalt+) to allow for comparison. We will interview up to 80 HCWs from Penn Medicine in order to understand the attitudes and decision-making involved in mental health strain during COVID-19. We will recruit across both arms of the Aim 1 study to investigate HCW in usual care Cobalt (n=40) and from those in the intervention arm Cobalt+ (n=40). Using data from Aim 1, we will use a deviance sampling approach to identify HCW from the top and bottom 25% percentiles of anxiety and depression scales. This deviant selection is intentional to maximize the potential to identify themes unique to high or low levels of mental health strain. Individual scale scoring will not be revealed in order to limit responder bias, increase response rates and avoid attribution bias. We will recruit HCWs using a series of e-mails (up to 4 email invitations) and text messages (up to 3 text invitations). We will contact participants to obtain consent, ensure study procedures are described, answer questions and offer a $50 gift card for their time. The goal is to conduct 80 audio-recorded semi-structured interviews (~20-30 minutes). Interview questions will be aligned with themes from our preliminary studies and focus groups with front line healthcare workers early in the pandemic. Participants will be contacted one month following the end of the Aim 1 intervention period and consented via WTH. Participants will not be informed as to why they were selected and confidentiality will be maintained. There is variability in how individuals experience anxiety, depression, stress and overall well-being. Understanding how HCWs think about these emotions, their physical and mental states of being, and the decisions to access and utilize mental health resources are essential to the design of interventions to facilitate and support the workforce. Understanding how HCWs approach mental health assessments for themselves and the stigma associated with care is also critical in designing programs that are acceptable and used. Investigation of the factors that impact mental health care in HCWs can be supported by semi-

structured interviews which ask open-ended questions and allow participants to describe key attitudes, decision-making processes, and emotions that are otherwise difficult to assess.

5.3. Study Duration

The RCT will compare this pull model with a new model, the intervention named Cobalt+, which will proactively reach out to and engage individuals in order to reduce barriers in identifying a need for and accessing mental health care. The intervention group will have access to the same Cobalt resources as those in the usual care group but will also receive a comprehensive suite of services including: 1) monthly automated text messaging reminders and links to Cobalt resources 2) intermittent mental health assessments (PHQ-9, GAD-7) at enrollment, month 2, month 4 and month 6 which triage individuals to an opt-out appointment with a resilience coach, psychotherapist, or psychiatrist based on their results. Of note, all employees will have access to Cobalt whether they are in the study or not. Through the proposed RCT we will evaluate whether the intervention, a proactive model of care delivery (Cobalt+) impacts the primary outcome measure (depression and anxiety) and secondary outcome measures (well-being, satisfaction with access to care, and measures of work productivity [e.g. absenteeism]).

5.4. Facilities

The research team is highly experienced and well-positioned to execute this project rapidly and across departments and hospitals within Penn Medicine. Team members have worked closely on many clinical and research programs, conducted clinical trials using mobile and digital tools and platforms, and conducted trials specifically in the target vulnerable population, HCW. Pilot work from this team has led to multiple peer-reviewed publications in wellbeing, mental health, and deploying digital tools and platforms. We have enthusiastic support from Penn Medicine and departmental leadership in developing strategies to identify, support, and protect HCW well-being and mental health. Our team is interwoven within the frontline clinical workforce. The research team brings together expertise in real-time patient- centered engagement using digital tools (Merchant, Agarwal, Asch), digital health (Merchant, Agarwal), well-being research (Bellini, Asch, Agarwal, Wolk), survey and qualitative methods (Shea), advanced biostatistics and computational methods (Mitra) and innovations in health systems (Asch, Merchant, Agarwal). Our team also has integral roles in Penn Medicine leadership which can help with project stewardship and uptake: PI (Merchant) serves as the Penn Medicine Associate Vice President of Digital Health, Co-I (Bellini) is the Senior Vice Dean for Academic Affairs and health system lead for Cobalt, and Co-I (Asch) is the Executive Director of the Penn Medicine Center for Health Care Innovation and Innovation lead for Cobalt. Additionally, Penn is an opportune venue to conduct COVID-19 related research as a leader in the region in acute and virtual care during the pandemic. This proposal represents a natural next step to build upon our expertise and track record of success in designing, conducting, and disseminating meaningful and actionable patient and provider centered research. Advisory Board and stakeholder engagement: To insure input from providers that will inform the research, we will convene an advisory board representing entities from each (n=12) of the direct patient care groups (e.g. physicians, nurses, certified nursing assistants, lab technicians, radiology technicians physical therapists, occupational therapists, pharmacists, pharmacy technicians, patient registration staff, patient intake coordinators, environmental service personnel). We have strong existing relationships with these groups and will meet with them before the study initiates and then every 6 months thereafter for input, engagement, and feedback. Some of the members of this group will also be recruitment champions identified. This step is critically important and we will work collaboratively with this group for stakeholder guidance about all aspects of execution and reporting for the study.

5.5. Key Inclusion Criteria

There are no inclusion and exclusion criteria for any subpopulation based on race/ethnicity, age, gender, status as a vulnerable population. Eligibility criteria for study enrollment will include: 1) age 18 years or older; 2) regular, daily access to a smartphone 3) able to communicate fluently in English (as the current Cobalt assessments and resources are in English at this time), 4) work at least 4 hours per week in either a hospital or outpatient based settings (e.g. physicians, nurses, certified nursing assistants, lab technicians, radiology technicians, physical therapist, occupational therapist, pharmacists, pharmacy technicians, patient registration staff, receptionists/patient intake coordinators, environmental service personnel) approximately 4 hours/week (on average 192 hours/ 48 weeks in a year) in the study time frame. Eligible participants will then have an opportunity to consent to study participation. Any HCW meeting these criteria will be eligible to participate in the research; we will not exclude any potential participant based on a priori criteria.

5.6. Key Exclusion Criteria

1). Under 18 years of age; 2) does not have an interest in participating in a 9-month study and willing to complete regular surveys; 3) does not have regular, daily access to an SMS-capable phone 4) unable to communicate fluently in English (as the current Cobalt assessments and resources are in English at this time), 5) does not provide direct patient care approximately 4 hours/week (on average 192 hours/ 48 weeks in a year) in the study time frame. 6). Not willing to provide informed consent

6. Subject Recruitment

6.1. Target Population

The population of interest for this proposal is HCWs who in the midst of COVID-19 are at increased risk for mental health symptoms and conditions. We will also utilize University groups and motivated mental healthcare champions to specifically promote inclusion of female and underrepresented minority HCW in the RCT and qualitative aim. This focus aligns with the RFA which identifies vulnerable populations as: medical personnel with direct patient care and NIH-designated health disparity racial/ ethnic minorities. We specifically include a broad definition of HCWs (e.g. physicians, nurses, certified nursing assistants, lab technicians, radiology technicians, physical therapists, occupational therapists, pharmacists, pharmacy technicians, patient registration staff, coroners, receptionists/patient intake coordinators, environmental service personnel) that are not routinely evaluated jointly and with the same intervention. While much of the attention in the pandemic has been on physicians and nurses we aim to focus on a broader group that is at risk for COVID-19 and mental health symptoms and conditions

6.2. Subjects at Penn

1,250

6.3. Accrual

To recruit potential study participants, we will send an email about the study to the entire Penn Medicine employee listserv, as these individuals are the same recipients of Penn Medicine news and Covid-19 updates who have received prior information about the availability of Cobalt. Emails will be sent up to 5 times to this employee list. The plan to outreach to employees in this manner has been approved by both Penn Medicine Human Resources and the Penn Medicine Chief Operation Officer. We will specifically engage recruitment champions in the study design and for support in extending the reach of the recruitment email to underrepresented minority HCWs. These recruitment champions will include each Departments Vice Chair for Diversity and Inclusion to support sending the email about the study to their Departments and groups within their Departments specifically focused on diversity and inclusion. We will also engage Centers, Departments, and Groups at Penn Medicine to support dissemination of the study announcement. These include the Office of Inclusion and Diversity, the Alliance of Minority Physicians, Penn Medicine Center for Health Equity Advancement, Bold Solutions

Initiative, FOCUS on health and leadership for women, Penn Promotes research on sex and gender in health, and the Program for Health Equity in Education and Research. Furthermore, we will recruit in-person across the Penn Medicine campus such as tabling outside of prominent buildings and departments (New Pavilion and cafeterias) and attend department morning huddles / meetings to accommodate staff members who have limited access to their email accounts. We will also be intentional about diversity of representation in the images in all study materials and highlight the diversity in our project team. We base our power considerations on baseline data presented in an RCT conducted by Graham et al. In their study, they report a mean baseline PHQ-9 of 14.0 (SE=5.0) and mean GAD-7 of 11.6 (SE=4.6). We assume a type I error rate of 2.5% (.05/2) to account for the two separate outcomes that we will be evaluating. We aim to enroll 1250 participants with the anticipation that there will be 20% attrition.

6.4. Patient Subject Recruitment

To recruit potential study participants, we will send an email about the study to the entire Penn Medicine employee listserv, as these individuals are the same recipients of Penn Medicine news and Covid-19 updates who have received prior information about the availability of Cobalt. Emails will be sent up to 5 times to this employee list. The plan to outreach to employees in this manner has been approved by both Penn Medicine Human Resources and the Penn Medicine Chief Operation Officer (see letters of support). Exploratory Aim1 specifically seeks to determine if the effect of the intervention varies across patient subgroups (e.g. race and gender) as we recognize the importance of evaluating the effect of Cobalt in these specific populations. We will specifically engage recruitment champions in the study design and for support in extending the reach of the recruitment email to underrepresented minority HCWs. These recruitment champions will include each Departments Vice Chair for Diversity and Inclusion to support sending the email about the study to their Departments and groups within their Departments specifically focused on diversity and inclusion. We will also engage Centers, Departments, and Groups at Penn Medicine to support dissemination of the study announcement. These include the Office of Inclusion and Diversity, the Alliance of Minority Physicians, Penn Medicine Center for Health Equity Advancement, Bold Solutions Initiative, FOCUS on health and leadership for women, Penn Promotes research on sex and gender in health, and the Program for Health Equity in Education and Research. We will also be intentional about diversity of representation in the images in all study materials and highlight the diversity in our project team. To promote retention, participants will be emailed up to 4 times and texted (up to three times) to nudge survey and assessment completion. For compensation, each participant will receive up to $200: $50 at enrollment, $75 at 6 months and $75 at 9 months for 3 total surveys. Submission for subject reimbursement will occur immediately upon completion of surveys and assessments and occur through the Way to Health platform which facilitates timely payment.

6.5. Subject Compensation

To promote retention, participants will be emailed up to 4 times and texted (up to three times) to nudge survey and assessment completion. For compensation, each participant will receive up to $200: $50 at enrollment, $75 at 6 months and $75 at 9 months for 3 total surveys. Submission for subject reimbursement will occur immediately upon completion of surveys and assessments and occur through the Way to Health platform which facilitates timely payment. Each participant will receive $50 for interview completion.

7. Study Procedures

7.1. Consent Process

To recruit potential study participants, we will send an email about the study to the entire Penn Medicine employee listserv, these individuals are the same recipients of Penn Medicine news and COVID-19 updates who have received prior information about the availability of Cobalt. Emails will be sent up to 4 times to this employee list. This proposal includes a letter of support from the Penn Medicine Director of Human Resources and Penn Medicine Chief Operation Officer indicating support for the project and access to these listservs. Participants will provide electronic informed consent before participation in the study. The electronic consent form is hosted on Way to Health, HIPAA Compliant Platform. Participants will be notified that enrollment is voluntary and declining to participate will not change their access to Cobalt or have an impact on their employment. Participants will be consented with ample time to discuss consent. Participants will be given contact information (including telephone and email) for the principal investigator (Dr. Merchant), and the University of Pennsylvania IRB to address any potential concerns. A detailed Manual of Operations will be developed for the study and procedures will be designed to ensure efficient and prompt updating. Our project is designed to function entirely remotely (recruitment, consent, enrollment, survey completion) All study participants read and review the informed consent document independently on Way to Health but research team members are readily available to discuss the form and answer questions via email or telephone communications, all noted on the instructions of the informed consent document on Way to Health. All investigators have experience in developing a manual of operations. All study staff will undergo a thorough training and certification procedure prior to protocol implementation, including CITI Course in the Protection of Human Research Participants online training in research ethics. The initial training will be conducted by the PI and will include an overview of the study, detailed explanation of the study protocol, and hands-on practice of specific protocol components. All study staff will be observed conducting randomly selected protocol duties at least twice annually and evaluated using specifically designed checklists.

7.1.1.1. Waiver or Alteration of Informed Consent

Not applicable

7.1.1.2. Minimal Risk

Since we are recruiting Penn Medicine employees, the informed consent highlights how their Penn Medicine employer, director, supervisor, or co-worker may be an investigator in this research study. The document also includes language on how if the individual chooses not to participate, there will be no loss of benefits to which they are otherwise entitled. Their employment is not in jeopardy based on their enrollment status. Given that the study compares the same process delivered as either a pull (usual care = Cobalt) or a push (intervention = Cobalt +) we believe risk is low. Nevertheless, we will engage a 3-member independent Data and Safety Monitoring Board to oversee the trial. In accordance with conventional practices, we will not identify members in advance so as not to disqualify potential proposal reviewers. All subjects will be consented for participation and will have the ability to withdraw at any time. There is minimal risk to the participants as Penn Cobalt will triage patients accordingly to level of services for mental health support. All qualitative results will be de-identified and any collected PHI data will be stored securely and separately. Participants can elect to not participate in the study without any consequences. Participants can also opt-out of participating at any time. All employees have access to Cobalt and associated services; participants in the intervention arm may be less likely to experience risks associated with the research because they are receiving pushed services designed to reduce barriers to access of mental health services.

7.1.1.3. Impact on Subject Rights and Welfare

We will be obtaining informed consent from patients who have been provided a thorough explanation of the study and the opportunity to ask any questions about study participation. Patients will be read the entire consent and given the option to participate.

7.1.1.4. Waiver Essential to Research

All participants will be recruited through remote procedures.

7.1.1.5. Written Statement of Research

This study does not operate under a written statement of research.

7.2. Procedures

We will conduct a prospective two-armed randomized control trial to test the effect of tailored mental health support on self-reported HCW mental health (anxiety and depression) during the response and recovery phases of the COVID-19 pandemic. The control group will have access to Cobalt which is usual care for all Penn Medicine employees. The Cobalt platform is web-based and is accessible from any device or desktop. Cobalt uses contextual surveys and evidence-based assessments to triage individual users to curated content and appropriate group or individual support. The current, and usual care, model is a digital version of what exists at other institutions where resources are available for staff to access wellness and mental health services. The usual care Cobalt model requires staff to personally initiate multiple steps: 1) identify a personal need for support, 2) know where to seek support and care, 3) schedule an appointment, and 4) attend the appointment. In this context, the individual has to pull the resources they need and there may be several barriers to completing each step. This is particularly relevant as mental health conditions can compromise insight, motivation, and decision making, thereby making self-directed engagement in care (pull) more challenging. The RCT will compare this pull model with a new model, the intervention named Cobalt+, which will proactively reach out to and engage individuals in order to reduce barriers in identifying a need for and accessing mental health care. The intervention group will have access to the same Cobalt resources as those in the usual care group but will also receive a comprehensive suite of services including: 1) monthly automated text messaging reminders and links to Cobalt resources 2) intermittent mental health assessments (PHQ-9, GAD-7) at enrollment, month 2, month 4 and month 6 which triage individuals to an opt-out appointment with a resilience coach, psychotherapist, or psychiatrist based on their results. Of note, all employees will have access to Cobalt whether they are in the study or not. Through the proposed RCT we will evaluate whether the intervention, a proactive model of care delivery (Cobalt+) impacts the primary outcome measure (depression and anxiety) and secondary outcome measures (well-being, satisfaction with access to care, and measures of work productivity [e.g. absenteeism]).

8. Analysis Plan

We will conduct an intent to treat (ITT) analysis. The ITT analysis will include all randomized participants in the groups to which they were randomly assigned. Baseline demographic and clinical characteristics will be reported as frequency and percent for categorical variables and median and distribution for continuous variables. We will compare baseline characteristics between intervention and control arms using t-tests for continuous variables and chi-squared tests for categorical variables. The goal of these comparisons will be to determine if the two arms are balanced on baseline variables after randomization. Our primary analysis will focus on the difference in PHQ-9 and GAD-7 scores at 6 months between the two arms. We will estimate and test differences in means between arms using two- sample t-tests and generalized linear models that account for baseline measurements of PHQ-9 and GAD-7. Missing data will be assessed for patterns and multiple imputation will be used if deemed appropriate. Baseline covariates that are found to be imbalanced between arms may be adjusted for in the model if they were deemed to be potential confounders a priori to adjust for potential confounding and for efficiency gain. We will use all available PHQ-9 and GAD-7 scores on eligible patients from randomization

through the last observation. A p0.05 will be deemed statistically significant but emphasis will be placed on point estimates and confidence intervals. Secondary outcomes of mental healthcare utilization, satisfaction with connection to services, and qualitative feedback on mobile engagement will also be modeled using GEE with mean models specified based on the distribution of the specific outcome (e.g. Poisson or negative binomial for number of mental healthcare visits, logit for binary responses). Potential associations between system-level or regional level case burden and PHQ-9 and GAD-7 scores at baseline and over-time will be assessed with generalized linear models and GEE. Exploratory Aim 1: Statistical approach: The statistical approach for studying HTE will be to test for the statistical interaction between the intervention and race and separately by gender in the GEE models described above. Although our study was not designed specifically to detect HTE, if 20% of our trial participants are Black (based on targeted recruitment efforts), our study will achieve 80% power (assuming type I error of 0.05) to detect a 1.8 difference in mean GAD-7 scores and a 2.0 difference in mean PHQ-9 scores between arms at 6 months. We anticipate that at least 50% of participants will be female. Hence, our study is powered to detect a 1.2 difference in mean GAD-7 scores and a 1.3 difference in mean PHQ-9 scores between arms at 6 months among women.

Aim 2: Power: no power calculation will be performed for this qualitative aim consistent with prior qualitative aims we will aim to interview 80 participants from Aim 1 and evaluate themes until saturation Analysis: Using thematic analysis, we will analyze HCW interview transcripts to identify recurring themes in attitudes and beliefs toward mental health, well-being, and mental health care or resources. Interview audio will be transcribed and uploaded into NVivo software (available as a Penn resource). In total we plan for up to 80 semi-structured interviews, or until inductive thematic saturation is reached in each group meaning no new themes are emerging. Analysis of qualitative, semi-structured interviews utilizes a series of systematic steps in transcribing, developing a code for thematic content, and subsequent coding of interviews. A modified grounded theory approach will be used to evaluate interview transcriptions. Analysis will begin shortly after the first few interviews are completed. An emergent, iterative open coding process will be followed and two trained coders from the research team will jointly read five transcripts and draft the outline of a codebook. Each coder will then independently code five more transcripts and adjust the codebook as needed. We will review the codes, resolve discrepancies and finalize coding procedures. Following adjudication of any areas of discrepancy, the research team coders will be assigned batches of transcripts for independent coding, sharing 25% of the transcripts in each batch for ongoing inter-rater agreement assessment. We will review coding for interrater reliability, measured by Cohens kappa (0.8), and overall comprehensiveness. If a high kappa is not achieved, further double coding and refining of the code will continue. This approach to coding and analyzing qualitative data has been widely used. Strategies used to ensure reliability and validity in the qualitative data will include a comprehensive audit trail, checks between coders, team debriefing, and corroboration of findings across participants.

9. Subject Confidentiality

The research team will exercise extreme caution with identifiable private information. Patients randomized to the intervention arm will receive weekly messages from Way to Health. In the patients informed consent, they are instructed and agree by signing the document that they will not disclose personal health information (PHI) on the Way to Health texting platform and only respond to the text message prompts. When patient participants (both control and intervention) text into the Way to Health or complete a survey, the research team will receive email notification immediately. The research team is instructed to review the message within 1-4 hours of delivery, Monday through Friday, 8 am to 5pm. The line will be closed on Saturday and Sunday however patient participants will receive an out of office

message if they text in; the messages notes, Thank you for reaching out, we will get back to you when we get back in the office! and a Patient Inquiry Incident is created. An exception to confidentiality is if significant suicidal ideation, is exhibited through text message or via PHQ-9. Question 9 asks about thoughts of being better off dead or of hurting oneself in some way over the last two weeks with an associated scale delineating not at all, several days, more than half the days, or nearly every day. Those indicating any response beyond not at all will be triaged to immediate access to a therapist via the Cobalt platform. This is the current standard of care and will be the same for both Cobalt and Cobalt plus in this trial.

10.1 Subject Privacy

Privacy refers to the person's desire to control access of others to themselves. Privacy concerns people, whereas confidentiality concerns data. Describe the strategies to protect privacy giving consideration to the following: The degree to which privacy can be expected in the proposed research and the safeguards that will be put into place to respect those boundaries. The methods used to identify and contact potential participants. The settings in which an individual will be interacting with an investigator. The privacy guidelines developed by relevant professions, professional associations and scholarly disciplines (e.g., psychiatry, genetic counseling, oral history, anthropology, psychology). Interview audio-recordings will be transcribed verbatim and all identifiers will be removed. The recording and transcript will be kept a secure and locked area with access limited to designated researchers. After we analyze the recordings, we will destroy recordings after data analysis or completion of the study. All encounters will take place online via BlueJeans or Way to Health, HIPAA compliant platform.

10.2 Data Disclosure

Survey data will be submitted to the National Institute of Mental Health Data Archive (NDA) at the National Institutes of Health (NIH). NDA is a large database where deidentified study data from many NIH studies are stored and managed. De-identified study data means that all personal information (such as name, address, birthdate and phone number) is removed and replaced with a code number. PHI will not be shared with anyone outside the parameters of the study as detailed in the Consent/HIPAA process.

10.3 Data confidentiality

The following methods will be employed to protect patient PHI for this research study:

x Computer-based files will only be made available to personnel involved in the study through the use of access privileges and passwords.

x Prior to access to any study-related information, personnel will be required to sign statements agreeing to protect the security and confidentiality of identifiable information.

x Wherever feasible, identifiers will be removed from study-related information.

x A Certificate of Confidentiality will be obtained, because the research could place the subject at risk of criminal or civil liability or cause damage to the subject's financial standing, employability, or liability.

x Precautions are in place to ensure the data is secure by using passwords and encryption, because the research involves web-based surveys.

x Audio and/or video recordings will be transcribed and then destroyed to eliminate audible identification of subjects.

11 Consent Process Overview

Participants will enroll in this study via a remote recruitment process. The study coordinator will review the consent script, which will include a description of the voluntary nature of participation, the study procedures, risks and potential benefits in detail. Participants will be told that all information will be kept strictly confidential, except as required by law. Subjects will be provided a copy of the consent document. All efforts will be made by study staff to ensure subject privacy. Enrollment will be conducted by the study coordinators who will enter patient information directly into the Way to Health platform once a participant has consented to participate.

11.2 Potential Study Risks

Given that the study compares the same process delivered as either a pull (usual care = Cobalt) or a push (intervention = Cobalt +) we believe risk is low. Nevertheless, we will engage a 3-member independent Data and Safety Monitoring Board to oversee the trial. In accordance with conventional practices, we will not identify members in advance so as not to disqualify potential proposal reviewers. All subjects will be consented for participation and will have the ability to withdraw at any time. There is minimal risk to the participants as Penn Cobalt will triage patients accordingly to level of services for mental health support. All qualitative results will be de-identified and any collected PHI data will be stored securely and separately. Participants can elect to not participate in the study without any consequences. Participants can also opt-out of participating at any time. All employees have access to Cobalt and associated services; participants in the intervention arm may be less likely to experience risks associated with the research because they are receiving pushed services designed to reduce barriers to access of mental health services..

11.3 Potential Study Benefits

All participants will have access to Cobalt, digital mental health and well-being platform which provides stepped care based on needs. Additionally, participants using either Cobalt or Cobalt+ may identify depression or anxiety otherwise unknown to them and then will be connected to care. The direct benefit to human subjects is in the use of Cobalt (usual care) and for those who are in the Cobalt+ intervention arm, access to a pushed model of care where resources are opt-out. The information learned from this study will hopefully help contribute to the fields of digital health and mental health. The risks are reasonable in relation to the importance of knowledge expected to be gained. Information learned from this study could improve our understanding of how digital platforms can be used to engage participants, measure mental health using validated scales, and connection individuals to resources and care. We will also gain insights about how these platforms impact access to care and perceived barriers to care in healthcare workers. Ultimately, the data can be used to better understand and improve strategies for protecting and supporting the mental health of healthcare workers throughout the COVID-19 pandemic and its associated phases, but also in the future.

11.4 Alternatives to Participation

Not participate in the study. As a reminder, all Penn Medicine and University of Pennsylvania have access to Cobalt resources, regardless of their participation in the study.

11.5 Data and Safety Monitoring

The study will be single-blinded and the study biostatistician will determine when to unblind the study. Given that the study compares the same process delivered as either a pull (usual care = Cobalt) or a push (intervention = Cobalt +) we believe risk is low. Nevertheless we will engage a 3-member independent Data and Safety Monitoring Board to oversee the trial. In accordance with conventional practices, we will not identify members in advance so as not to disqualify potential proposal reviewers. Given that the study compares the same process delivered as either a pull (usual care) or a push (intervention = well-being platform) we believe risk is low. Nevertheless, we will engage a 3-member independent Data and Safety Monitoring Board to oversee the trial. In accordance with conventional practices, we will not identify members in advance so as not to disqualify potential proposal reviewers. These three external investigators will have expertise in mental health and wellness, employee health, and digital health. In accordance with conventional practices, we will not identify members in advance so as not to disqualify potential proposal reviewers. The responsibilities of the DSMB will include: Reviewing the research protocol, consent, and plans for DSMB Evaluate the progress of the trial: recruitment, retention, data quality, and adverse events Protection of participant safety Ensure high standards for privacy and no breech of data security Provide written summary reports to the PI (Merchant) and investigators after convening Provide a written summary report to the NIH funding body if required This study will not have pre-specified stopping rules. The study will be single-blinded and the study biostatistician will determine when to unblind the study..

Final Study Protocol

1. **Abstract**……17
2. **Background**……17
3. **Overall Objectives**……19
3.1. Primary Aims……19
3.2. Secondary Aims……19
4. **Primary Outcome Variable**……19
5. **Secondary Outcome Variable(s)** ……19
6. **Study Design**……20
6.1. Phase……20
6.2. Design……20
6.3. Study Duration……21
6.4. Facilities……22
6.5. Key Inclusion Criteria……22
6.6. Key Exclusion Criteria……22
7. **Subject Recruitment**……23
7.1. Target Population……23
7.2. Subjects at Penn……23
7.3. Accrual……23
7.4. Patient Subject Recruitment……24
7.5. Subject Compensation……25
8. **Study Procedures**……25
8.1. Consent Process……25
8.1.1. Waiver of Alteration of Informed Consent……25
8.1.2. Minimal Risk……25
8.1.3. Impact on Subject Rights and Welfare……26
8.1.4. Waiver Essential to Research……26
8.1.5. Written Statement of Research……26
8.2. Procedures……26
9. **Analysis Plan**……26
10. **Subject Confidentiality**……27
10.1. Subject Privacy……27
10.2. Data Disclosure ……28
10.3. Data confidentiality……28
11. **Consent Process Overview**……**29**
11.1. Potential Study Risks……29
11.2. Potential Study Benefits……29
11.3. Alternatives to Participation……30
11.4. Data Safety and Monitoring ……30

1. Abstract

Early in the pandemic, our team developed and implemented Penn Cobalt across the University of Pennsylvania Health System which spans six acute-care hospitals and employs over 43,000 individuals. Cobalt is a web-based platform that curates mental health and wellness content and provides connection to group and individual support. Using validated mental health assessments, Cobalt triages users to the right level and type of support across three categories: In the studio synchronous group sessions, On your time asynchronous content, and 1:1 support. The 1:1 support represents a stepped care model of mental health services and includes peer support, resilience coaches offering psychological first aid, psychotherapists and psychiatrists. Cobalts embedded scheduling and telehealth capabilities also provide HIPAA-compliant mental health care in a convenient, patient-centered model. Cobalt content continuously adapts and evolves through crowdsourced feedback to encompass locally defined and sensitive resources (e.g. addressing racial trauma). Over the first 7 months, Cobalt has had over 140,000-page views and 18,300 unique users engaging with its content and support. The platform identified 111 HCWs reporting thoughts of self-harm and connected those individuals with a mental health provider for support and evaluation within 24 hours.

2. Background

Epidemics are often associated with significant mental health consequences to society. Large-scale disasters, whether traumatic (e.g., the World Trade Center attacks or mass shootings), natural (e.g., hurricanes), or environmental (e.g., Deepwater Horizon oil spill), are often accompanied by increases in depression, posttraumatic stress disorder (PTSD), substance use disorder, and a broad range of other mental health and behavioral disorders, domestic violence, and child abuse. For example, prior work showed that 5% of the population affected by Hurricane Ike in 2008 met the criteria for major depressive disorder in the month after the hurricane; 1 out of 10 adults in New York City showed signs of the disorder in the month following the 9/11 attacks. And almost 25% of New Yorkers reported increased alcohol use after the tragic attacks. Communities affected by the Deepwater Horizon oil spill showed signs of clinically significant depression and anxiety. The SARS epidemic was also associated with increases in PTSD, stress, and psychological distress in patients and clinicians. For such events, the impact on mental health can occur in the immediate aftermath and then persist over long time periods. The rates of stress and burnout were high in health care workers prior to the pandemic and those rates have persisted or increased. In March of 2020, COVID-19 emerged as a global pandemic resulting in acute stressors directly impacting an already vulnerable and strained health care system. Healthcare workers (HCWs) are facing unique challenges during the COVID-19 pandemic related to rapid shifts in care, strains on availability of personal protective equipment, moral injury, and concerns about risks of infection. The challenges confronting HCWs continue outside of work including childcare responsibilities, career trajectory and concern for employment status with dynamically shifting practice models and patient volumes. The mental health burdens of frontline HCWs began to emerge in early retrospective studies from China and Italy; with early reports of increases in clinician suicide due to the pandemic. HCWs are predicted to face repeated acute triggering events throughout the long period of disillusionment we now find ourselves in as we slowly inch towards reconstruction and recovery with the COVID-19 pandemic. Additional HCW stress is associated with the real or perceived risk of contracting COVID-19 or spreading it to loved ones. A 2020 MMWR report on occupation type and job setting of HCWs with COVID-19 showed that the relative percentage of cases was high in nurses (1742, 29.5%), environmental services (EVS) (330, 5.6%), physicians (190,3.2), and respiratory therapists (44, 0.7%). COVID-19 has also affected HCWs across demographics with higher rates of death in underrepresented minorities. Among HCW with COVID-19 the median age was 41 years and 79% were females. For the 69,678 (69%) of cases with available data on race and ethnicity the distribution was Whites (47%), Blacks (26%), Hispanics (12%) Asians (9%). Compared with COVID-19 survivors, non-

survivors were more likely to be older (median age 62), male (38% vs. 22%), Asian (20% vs. 9%), or Black (32% versus 25%). The increased exposure to COVID-19 may also limit physical contact of HCW with usual support networks and systems (e.g. family or faith-based groups) or lead to increased stress because of home based caregiving for elderly or immunocompromised family members. HCWs identifying as Black, indigenous, or of color are a vulnerable population. Prior reports support that black adults are approximately 10% more likely to report psychological distress when compared with white adults. Data from the Health and Human Services Office of Minority Health also shows that black adults are more likely than white adults to report hopelessness, sadness, and feeling like everything is an effort. There are significant barriers to accessing mental health care for black adults and it is estimated that only one in three black adults who need mental health care receive it. Many reasons contribute to these disparities including: costs of care, stigma associated with care, provider bias, lack of knowledge regarding available mental health therapies, and the lack of minority mental health professionals. For those who do receive care, the care is often of lower quality and not culturally aligned. Black HCWs have historically been subject to racism, discrimination, and inequity--and the national events centered around racial injustice coupled with COVID-19 have likely exacerbated mental health and well-being in measurable and sustained ways. Minority HCWs were already strained, often working within settings where they themselves are subject to microaggressions, overt acts of racism, structural racism and are direct observers of racial inequities in patient care leading to significant disparities in morbidity and mortality. Racial minorities are also disproportionately in jobs with high exposure risk including grocery stores, public transportation, factories, and health care facilities. A meta-analysis of 293 studies identified that racism significantly contributed to worse mental and physical health. Based on findings from more than 60 structured interviews with Black HCWs, the noted sociologist Adia Wingfield summarized her findings and noted: being a Black health care worker comes with specific difficulties that can easily go unnoticed. In a pandemic where Black populations are among the hardest hit, these difficulties are likely being magnified exponentially. Health care systems are undoubtedly taxed, but in the interest of their workers, they should consider ways they can support Black health care providers to offset the kinds of burnout and stresses research indicates they are likely experiencing right now. In this national emergency, health care systems may need to think past providing health care just for patients and consider the health of their workers, perhaps through counseling and support groups, heeding employees suggestions for how systems can be improved. The social and economic consequences of COVID-19 are predicted to affect women differently than men. Prior reports support that women have been more likely to lose their jobs or leave them during the pandemic due to a need to provide child and elder care as well as to support home schooling. While this unpaid labor has increased, paid work opportunities have also decreased for women. Increases in sexual and physical violence toward women during lockdowns have also been reported. The United Nations has identified increased vulnerability of female frontline workers and the need for targeted programs to specifically address their unique mental health needs due to a multitude of stressors. Prior reports have also focused on female frontline providers and physician researchers and threats to their professional careers, particularly academic, advancement due to barriers in work productivity as a result of COVID-19. The psychological impact of the delay in academic career progression (e.g. fewer grant submissions, fewer peer-reviewed publications, fewer leadership opportunities) could be pronounced and long standing. Considering how the pandemic has led to considerable strain on women and the unique impact on women in health care, this proposal specifically focuses on evaluating if Cobalt+ will have a greater impact on women and can serve as a platform for addressing inequities in mental health care for this vulnerable population.
COVID-19 has increased the use and application of digital technologies in health care including text messaging, mobile surveys, and telemedicine. Evolving and rapidly adopted approaches in mobile engagement through digital technology create scalable opportunities to assess individuals needs in real-time and can be used to rapidly deploy tailored well-being and mental health interventions. This

approach also offers a strategy to reveal the central tendencies, distributions, and associations which, in population-based studies, can immediately inform a health systems approach toward maintaining a healthy workforce and lowering barriers to mental health care.

3. Overall Objectives

Through a RCT, evaluate the effectiveness of Cobalt+ on HCW depression/anxiety (primary outcome) compared with Cobalt (usual care). *Aims*

3.1 Primary Aims

Aim 1: Through a RCT, evaluate the effectiveness of Cobalt+ on HCW depression/anxiety (primary outcome) compared with Cobalt (usual care).

Aim 2: To better understand perceptions of access to mental health care and the effectiveness of Cobalt compared with Cobalt+ among HCWs through semi-structured qualitative interviews.

3.2 Secondary Aims

Secondary outcomes will include well-being, satisfaction with access to care, and measures of work productivity. HTE will be explored for race and gender.

4. Primary Outcome Variable

Measures were selected based on clinical relevance, validity in the HCW population, and meaningfulness to the target population. To facilitate data sharing, measures below were also identified to align with the NIMH data archive when possible. The primary outcome will be depression and anxiety as measured by the Patient Health Questionnaire (PHQ-9) and General Anxiety Disorder (GAD-7) respectively. PHQ-9 and GAD-7 have been validated and used in multiple studies and provide easy, simple scales to quickly assess for depression and anxiety. PHQ9 is a 9-item score which ranges from 0 (least depressed) to 27 (most depressed. GAD-7 is a 7- item scale which assess the frequency of anxiety symptoms over the past two weeks on a 4-point Likert-scale ranging from 0 (never) to 3 (nearly every day). The total score of GAD-7 ranged from 0 to 21, with increasing scores indicating more severe functional impairments as a result of anxiety.

5. Secondary Outcome Variable(s)

Well-being index: The Well-being index (WBI) is a nine-question survey validated for use in HCW populations and considered important to health systems in managing the well-being of their workforce.

The World Health Organization- Five Well-Being Index (WHO-5) is a short self-reported measure of current mental wellbeing. The WHO-5 has been found to have adequate validity in screening for depression and in measuring outcomes in clinical trials. Item response theory analyses in studies of younger persons and elderly persons indicate that the measure has good construct validity as a unidimensional scale measuring well-being in these populations (Winther Topp et al., 2015).

Work productivity: To evaluate work productivity, we will use the Lam Employment Absence and Productivity Scale, or LEAPS. It is a 10-item, self-rated scale that takes only 3 to 5 minutes for the patient to complete. It is simple and easy to use. The items were chosen based on the symptoms that have the most impact on work productivity and the most common productivity problems experienced by patients with depression. The LEAPS was recently validated in a sample of 234 consecutive working patients meeting DSM-IV criteria for MDD attending a mood disorders outpatient clinic (Lam et al, 2009).

Satisfaction with care: To assess satisfaction we used the Satisfaction Index- Mental Health (SIMH)(Nabati, Shea, McBride, Gavin, & Bauer, 1998). The SIMH is a 12-item, self-report instrument developed to measure patient satisfaction with mental health care (Nabati, et al., 1998). The SIMH has high internal consistency reliability (Cronbachs alpha = 0.90), test- retest reliability (r = 0.79, p = 0.05), and sensitivity to change (Nabati, et al., 1998).

Perceived System usability: System Usability Scale (SUS) provides a quick and dirty, reliable tool for measuring the usability. It consists of a 10-item questionnaire with five response options for respondents; from Strongly agree to Strongly disagree. Originally created by John Brooke in 1986, it allows you to evaluate

a wide variety of products and services, including hardware, software, mobile devices, websites and applications. As required by the NIMH, WHODAS and DSM-5 Level 1 Cross-Cutting Symptom Measure are asked only at the month 9 follow-up survey. These questions are voluntary to complete. WHODAS is a 12-item questionnaire asks about difficulties due to health conditions. Health conditions include diseases or illnesses, other health problems that may be short or long lasting, injuries, mental or emotional problems, and problems with alcohol or drugs. Think back over the past 30 days and answer these questions, thinking about how much difficulty you had doing the following activities. For each question, please circle only one response. DOI: 10.2340/16501977-2583 The DSM-5 Level 1 Cross-Cutting Symptom Measure is a self- or informant-rated measure that assesses mental health domains that are important across psychiatric diagnoses. It is intended to help clinicians identify additional areas of inquiry that may have significant impact on the individuals treatment and prognosis. In addition, the measure may be used to track changes in the individuals symptom presentation over time. This version asks adults to consider how much (or how often) they have been bothered by specific problems in the past two weeks. https://doi.org/10.1176/appi.books.9780890425596 DSM-5 includes sensitive questions around as suicide ideation, mental health disorders including depression, anxiety, psychosis, dissociation and personality functioning, and tobacco, alcohol, and substance use.

6. Study Design

6.1 Phase

Not applicable

6.2 Design

We will conduct a prospective two-armed randomized control trial to test the effect of tailored mental health support on self-reported HCW mental health (anxiety and depression) during the response and recovery phases of the COVID-19 pandemic. The control group will have access to Cobalt which is usual care for all Penn Medicine employees. The Cobalt platform is web-based and is accessible from any device or desktop. Cobalt uses contextual surveys and evidence-based assessments to triage individual users to curated content and appropriate group or individual support. The current, and usual care, model is a digital version of what exists at other institutions where resources are available for staff to access wellness and mental health services. The usual care Cobalt model requires staff to personally initiate multiple steps: 1) identify a personal need for support, 2) know where to seek support and care, 3) schedule an appointment, and 4) attend the appointment. In this context, the individual has to pull the resources they need and there may be several barriers to completing each step. This is particularly relevant as mental health conditions can compromise insight, motivation, and decision making, thereby making self-directed engagement in care (pull) more challenging. The study will be single-blinded and the study biostatistician will determine when to unblind the study. Given that the study compares the same process delivered as either a pull (usual care = Cobalt) or a push (intervention = Cobalt +) we believe risk is low. Nevertheless, we will engage a 3-member independent Data and Safety Monitoring Board to oversee the trial. In accordance with conventional practices, we will not identify members in advance so as not to disqualify potential proposal reviewers. To assess factors that may contribute toward accessing and using mental health resources between Cobalt versus Cobalt+, we will conduct semi-structured interviews (telephone or virtual video conference) with HCWs enrolled in Aim 1. Standardized interview guides will be created using the consolidated framework for implementation research ((https://cfirguide.org/guide/app/#/guide_select) and thematic areas identified in literature review to ensure uniformity in how and what questions are asked of each group. Some questions within the guides will be the same between those in the traditional care arm (Cobalt) versus intervention (Cobalt+) to allow for comparison. We will interview up to 80 HCWs from Penn Medicine in order to understand the attitudes and decision-making involved in mental health strain during COVID-19. We will recruit across both arms of the Aim 1 study to investigate HCW in usual care Cobalt (n= up to 40) and

from those in the intervention arm Cobalt+ (n=up to 40). Using data from Aim 1, we will use a deviance sampling approach to identify HCW from the top and bottom 25% percentiles of anxiety and depression scales from month 6 to baseline. This deviant selection is intentional to maximize the potential to identify themes unique to high or low levels of mental health strain. Individual scale scoring will not be revealed in order to limit responder bias, increase response rates and avoid attribution bias. We intend Aim 2 study sample to be similar to the Aim 1 gender and race breakdown. Since we oversampled Black participants in Aim 1, we intend to have at least 25% Black participants in Aim 2. We will recruit HCWs using a series of e-mails (up to 4 email invitations) and text messages (up to 3 text invitations). We will contact participants to obtain consent, ensure study procedures are described, answer questions and offer a $50 gift card for their time. The goal is to conduct up to 80 audio-recorded semi-structured interviews (~20-30 minutes). Interview questions will be aligned with themes from our preliminary studies and focus groups with front line healthcare workers early in the pandemic. Participants will be contacted one month following the end of the Aim 1 study period and consented via WTH. Participants will not be informed as to why they were selected and confidentiality will be maintained. There is variability in how individuals experience anxiety, depression, stress and overall well-being. Understanding how HCWs think about these emotions, their physical and mental states of being, and the decisions to access and utilize mental health resources are essential to the design of interventions to facilitate and support the workforce. Understanding how HCWs approach mental health assessments for themselves and the stigma associated with care is also critical in designing programs that are acceptable and used. Investigation of the factors that impact mental health care in HCWs can be supported by semi-structured interviews which ask open-ended questions and allow participants to describe key attitudes, decision-making processes, and emotions that are otherwise difficult to assess. All Aim 2 participants will be informed that, You do not have to participate in any research study offered by your director, supervisor, or co-worker. If you choose not to participate, there will be no loss of benefits to which you are otherwise entitled. Your employment is not in jeopardy should you decide not to participate If you are interested in participating, A research team member is readily available to discuss the informed consent form with you and answer questions via email or telephone communications. You are free to decline or stop participation at any time during or after the initial consenting process. Please text back the Way to Health number or email digitalhealth@uphs.upenn.edu.The University of Pennsylvania has a significant financial interest in the Penn Medicine COBALT resources and delivery system used as a part of this protocol. It was developed by inventors at Penn Medicine, and if it were to be successful, the University of Pennsylvania will likely receive significant financial benefit. As a reminder, all Penn Medicine employees have access to COBALT for resources regardless of if they participate in the study. You are free to decline or stop participation at any time during or after the initial consenting process. To decline or stop participation, please email digitalhealth@uphs.upenn.edu or text in Bye to the Way to Health number. If Aim 2 participants expresses potentially sensitive information like mental health information, at the end of the interview, we will share free and confidential health resources.

6.3 *Study Duration*

The RCT will compare this pull model with a new model, the intervention named Cobalt+, which will proactively reach out to and engage individuals in order to reduce barriers in identifying a need for and accessing mental health care. The intervention group will have access to the same Cobalt resources as those in the usual care group but will also receive a comprehensive suite of services including: 1) monthly automated text messaging reminders and links to Cobalt resources 2) intermittent mental health assessments (PHQ-9, GAD-7) at enrollment, month 2, month 4 and month 6 which triage individuals to an opt-out appointment with a resilience coach, psychotherapist, or psychiatrist based on their results. Of note, all employees will have access to Cobalt whether they are in the study or not. Through the proposed RCT we will evaluate whether the intervention, a proactive model of care delivery (Cobalt+) impacts the primary outcome measure (depression and anxiety) and secondary outcome

measures (well-being, satisfaction with access to care, and measures of work productivity [e.g. absenteeism]). The study duration is approximately 29 months.

6.4 Facilities

The research team is highly experienced and well-positioned to execute this project rapidly and across departments and hospitals within Penn Medicine. Team members have worked closely on many clinical and research programs, conducted clinical trials using mobile and digital tools and platforms, and conducted trials specifically in the target vulnerable population, HCW. Pilot work from this team has led to multiple peer-reviewed publications in wellbeing, mental health, and deploying digital tools and platforms. We have enthusiastic support from Penn Medicine and departmental leadership in developing strategies to identify, support, and protect HCW well-being and mental health. Our team is interwoven within the frontline clinical workforce. The research team brings together expertise in real-time patient- centered engagement using digital tools (Merchant, Agarwal, Asch), digital health (Merchant, Agarwal), well-being research (Bellini, Asch, Agarwal, Wolk), survey and qualitative methods (Shea), advanced biostatistics and computational methods (Mitra) and innovations in health systems (Asch, Merchant, Agarwal). Our team also has integral roles in Penn Medicine leadership which can help with project stewardship and uptake: PI (Merchant) serves as the Penn Medicine Associate Vice President of Digital Health, Co-I (Bellini) is the Senior Vice Dean for Academic Affairs and health system lead for Cobalt, and Co-I (Asch) is the Executive Director of the Penn Medicine Center for Health Care Innovation and Innovation lead for Cobalt. Additionally, Penn is an opportune venue to conduct COVID-19 related research as a leader in the region in acute and virtual care during the pandemic. This proposal represents a natural next step to build upon our expertise and track record of success in designing, conducting, and disseminating meaningful and actionable patient and provider centered research.
Advisory Board and stakeholder engagement: To insure input from providers that will inform the research, we will convene an advisory board representing entities from each (n=12) of the direct patient care groups (e.g. physicians, nurses, certified nursing assistants, lab technicians, radiology technicians physical therapists, occupational therapists, pharmacists, pharmacy technicians, patient registration staff, patient intake coordinators, environmental service personnel). We have strong existing relationships with these groups and will meet with them before the study initiates and then every 6 months thereafter for input, engagement, and feedback. Some of the members of this group will also be recruitment champions identified. This step is critically important and we will work collaboratively with this group for stakeholder guidance about all aspects of execution and reporting for the study.

6.5 Key Inclusion Criteria

There are no inclusion and exclusion criteria for any subpopulation based on race/ethnicity, age, gender, status as a vulnerable population. Eligibility criteria for study enrollment will include: 1) age 18 years or older; 2) regular, daily access to a smartphone 3) able to communicate fluently in English (as the current Cobalt assessments and resources are in English at this time), 4) work at least 4 hours per week in either a hospital or outpatient based settings (e.g. physicians, nurses, certified nursing assistants, lab technicians, radiology technicians, physical therapist, occupational therapist, pharmacists, pharmacy technicians, patient registration staff, receptionists/patient intake coordinators, environmental service personnel) approximately 4 hours/week (on average 192 hours/ 48 weeks in a year) in the study time frame. Eligible participants will then have an opportunity to consent to study participation. Any HCW meeting these criteria will be eligible to participate in the research; we will not exclude any potential participant based on a priori criteria.

6.6 Key Exclusion Criteria

1). Under 18 years of age; 2) does not have regular, daily access to a smartphone 3) unable to communicate fluently in English (as the current Cobalt assessments and resources are in English at this

time), 4) does not not work at least 4 hours per week in either a hospital or outpatient based setting, 5). Not willing to sign the informed consent document.

7. Subject Recruitment

7.1 Target Population

The population of interest for this proposal is HCWs who in the midst of COVID-19 are at increased risk for mental health symptoms and conditions. We will also utilize University groups and motivated mental healthcare champions to specifically promote inclusion of female and underrepresented minority HCW in the RCT and qualitative aim. This focus aligns with the RFA which identifies vulnerable populations as: medical personnel with direct patient care and NIH-designated health disparity racial/ ethnic minorities. We specifically include a broad definition of HCWs (e.g. physicians, nurses, certified nursing assistants, lab technicians, radiology technicians, physical therapists, occupational therapists, pharmacists, pharmacy technicians, patient registration staff, coroners, receptionists/patient intake coordinators, environmental service personnel) that are not routinely evaluated jointly and with the same intervention. While much of the attention in the pandemic has been on physicians and nurses we aim to focus on a broader group that is at risk for COVID-19 and mental health symptoms and conditions. our study samples demographic information, specifically gender, race, and ethnicity compared to the larger employee group. We will receive de-identified proportions of this information from University of Pennsylvania administration. Demographic information will include: % female, % male, % non-reported/missing, % American Indian/Alaskan Native, % Asian, % Black or African American, % Native Hawaiian/Pacific Islander, % White, % I do not wish to answer, % non-reported/ missing, % Hispanic / Latinx, and % non-reported/missing. The demographic information would be used for internal discussions only with the Penn Medicine research personnel listed on our IRB HSERA protocol (IRB #: 848844).

7.2 Subjects at Penn 1,275

7.3 Accrual

To recruit potential study participants, we will send an email about the study to the entire Penn Medicine employee listserv, as these individuals are the same recipients of Penn Medicine news and Covid-19 updates who have received prior information about the availability of Cobalt. Emails will be sent up to 5 times to this employee list. The plan to outreach to employees in this manner has been approved by both Penn Medicine Human Resources and the Penn Medicine Chief Operation Officer. We will specifically engage recruitment champions in the study design and for support in extending the reach of the recruitment email to underrepresented minority HCWs. These recruitment champions will include each Departments Vice Chair for Diversity and Inclusion to support sending the email about the study to their Departments and groups within their Departments specifically focused on diversity and inclusion. We will also engage Centers, Departments, and Groups at Penn Medicine to support dissemination of the study announcement. These include the Office of Inclusion and Diversity, the Alliance of Minority Physicians, Penn Medicine Center for Health Equity Advancement, Bold Solutions Initiative, FOCUS on health and leadership for women, Penn Promotes research on sex and gender in health, and the Program for Health Equity in Education and Research. Furthermore, we will recruit in-person across the Penn Medicine campus such as tabling outside of prominent buildings and departments (New Pavilion and cafeterias) and attend department morning huddles / meetings to accommodate staff members who have limited access to their email accounts. We will also be intentional about diversity of representation in the images in all study materials and highlight the diversity in our project team. We base our power considerations on baseline data presented in an RCT conducted by Graham et al.92 In their study, they report a mean baseline PHQ-9 of 14.0 (SE=5.0) and mean GAD-7 of 11.6 (SE=4.6). We assume a type I error rate of 2.5% (.05/2) to account for the two

separate outcomes that we will be evaluating. We aim to enroll 1275 participants with the anticipation that there will be 27.5% attrition.

7.4 Patient Subject Recruitment

To recruit potential study participants, we will send an email about the study to the entire Penn Medicine employee listserv, as these individuals are the same recipients of Penn Medicine news and Covid-19 updates who have received prior information about the availability of Cobalt. Emails will be sent up to 5 times to this employee list. The plan to outreach to employees in this manner has been approved by both Penn Medicine Human Resources and the Penn Medicine Chief Operation Officer (see letters of support). Exploratory Aim1 specifically seeks to determine if the effect of the intervention varies across patient subgroups (e.g. race and gender) as we recognize the importance of evaluating the effect of Cobalt in these specific populations. We will specifically engage recruitment champions in the study design and for support in extending the reach of the recruitment email to underrepresented minority HCWs. These recruitment champions will include each Departments Vice Chair for Diversity and Inclusion to support sending the email about the study to their Departments and groups within their Departments specifically focused on diversity and inclusion. We will also engage Centers, Departments, and Groups at Penn Medicine to support dissemination of the study announcement. These include the Office of Inclusion and Diversity, the Alliance of Minority Physicians, Penn Medicine Center for Health Equity Advancement, Bold Solutions Initiative, FOCUS on health and leadership for women, Penn Promotes research on sex and gender in health, and the Program for Health Equity in Education and Research. Furthermore, we will recruit in-person across the Penn Medicine campus such as tabling outside of prominent buildings and departments (New Pavilion and cafeterias) and attend department morning huddles / meetings to accommodate staff members who have limited access to their email accounts. We will also be intentional about diversity of representation in the images in all study materials and highlight the diversity in our project team. For enrolled study participants, there is no email or text message follow for nudge messages. However, there is one text message or email follow up message (based on the participants communication preference) to complete survey assessments. For example, the message notes: Hi PARTICIPANT_FIRSTNAME, when you have a chance, please complete this short well-being survey: It only takes about 2 minutes! Thanks for your reply! For 6 and 9 month follow up survey reminders, we will send the following text message or email, Hi PARTICIPANT_FIRSTNAME, please complete the last follow up survey by clicking here: Once you complete it, $75 will be loaded on your ClinCard account. To promote retention, participants will be emailed up to 4 times and texted (up to three times) to nudge survey and assessment completion. For compensation, each participant will receive up to $200: $50 at enrollment, $75 at 6 months and $75 at 9 months for 3 total surveys. Submission for subject reimbursement will occur immediately upon completion of surveys and assessments and occur through the Way to Health platform which facilitates timely payment. Using data from Aim 1, we will use a deviance sampling approach to identify HCW from the top and bottom 25% percentiles of anxiety and depression scales. This deviant selection is intentional to maximize the potential to identify themes unique to high or low levels of mental health strain. Individual scale scoring will not be revealed in order to limit responder bias, increase response rates and avoid attribution bias. We will recruit participants enrolled in Aim 1 (RCT), using a series of e-mails (up to 4 email invitations) and text messages (up to 3 text invitations). We will contact participants to obtain informed consent for the interview to ensure study procedures are described, answer questions and offer a $50 gift card for their time. The goal is to conduct 80 audio-recorded semi-structured interviews (~20-30 minutes). Interview questions will be aligned with themes from our preliminary studies and focus groups with front line healthcare workers early in the pandemic. Participants will be contacted one month following the end of the Aim 1 study period and consented via

Way to Health. Participants will not be informed as to why they were selected, and confidentiality will be maintained.

7.5 Subject Compensation

To promote retention, participants will be emailed up to 4 times and texted (up to three times) to nudge survey and assessment completion. For compensation, each participant will receive up to $200: $50 at enrollment, $75 at 6 months and $75 at 9 months for 3 total surveys. Submission for subject reimbursement will occur immediately upon completion of surveys and assessments and occur through the Way to Health platform which facilitates timely payment. Each participant will receive $50 for interview completion.

8. Study Procedures

8.1 Consent Process

To recruit potential study participants, we will send an email about the study to the entire Penn Medicine employee listserv, these individuals are the same recipients of Penn Medicine news and COVID-19 updates who have received prior information about the availability of Cobalt. Emails will be sent up to 4 times to this employee list. This proposal includes a letter of support from the Penn Medicine Director of Human Resources and Penn Medicine Chief Operation Officer indicating support for the project and access to these listservs. Participants will provide electronic informed consent before participation in the study. The electronic consent form is hosted on Way to Health, HIPAA Compliant Platform. Participants will be notified that enrollment is voluntary and declining to participate will not change their access to Cobalt or have an impact on their employment. Participants will be consented with ample time to discuss consent. Participants will be given contact information (including telephone and email) for the principal investigator (Dr. Merchant), and the University of Pennsylvania IRB to address any potential concerns. A detailed Manual of Operations will be developed for the study and procedures will be designed to ensure efficient and prompt updating. Our project is designed to function entirely remotely (recruitment, consent, enrollment, survey completion) All study participants read and review the informed consent document independently on Way to Health but research team members are readily available to discuss the form and answer questions via email or telephone communications, all noted on the instructions of the informed consent document on Way to Health. All investigators have experience in developing a manual of operations. All study staff will undergo a thorough training and certification procedure prior to protocol implementation, including CITI Course in the Protection of Human Research Participants online training in research ethics. The initial training will be conducted by the PI and will include an overview of the study, detailed explanation of the study protocol, and hands-on practice of specific protocol components. All study staff will be observed conducting randomly selected protocol duties at least twice annually and evaluated using specifically designed checklists.

8.1.1.1 Waiver or Alteration of Informed Consent

Not applicable

8.1.1.2 Minimal Risk

Since we are recruiting Penn Medicine employees, the informed consent highlights how their Penn Medicine employer, director, supervisor, or co-worker may be an investigator in this research study. The document also includes language on how if the individual chooses not to participate, there will be no loss of benefits to which they are otherwise entitled. Their employment is not in jeopardy based on their enrollment status. Given that the study compares the same process delivered as either a pull (usual care = Cobalt) or a push (intervention = Cobalt +) we believe risk is low. Nevertheless, we will engage a 3-member independent Data and Safety Monitoring Board to oversee the trial. In accordance with conventional practices, we will not identify members in advance so as not to disqualify potential proposal reviewers. All subjects will be consented for participation and will have the ability to withdraw at any time. There is minimal risk to the participants as Penn Cobalt will triage patients accordingly to level of services for mental health support. All qualitative results will be de-identified and any collected

PHI data will be stored securely and separately. Participants can elect to not participate in the study without any consequences. Participants can also opt-out of participating at any time. All employees have access to Cobalt and associated services; participants in the intervention arm may be less likely to experience risks associated with the research because they are receiving pushed services designed to reduce barriers to access of mental health services.

8.1.1.3 Impact on Subject Rights and Welfare

We will be obtaining informed consent from patients who have been provided a thorough explanation of the study and the opportunity to ask any questions about study participation. Patients will be read the entire consent and given the option to participate.

8.1.1.4 Waiver Essential to Research

All participants will be recruited through remote procedures.

8.1.1.5 Written Statement of Research

This study does not operate under a written statement of research.

8.2 Procedures

We will conduct a prospective two-armed randomized control trial to test the effect of tailored mental health support on self-reported HCW mental health (anxiety and depression) during the response and recovery phases of the COVID-19 pandemic. The control group will have access to Cobalt which is usual care for all Penn Medicine employees. The Cobalt platform is web-based and is accessible from any device or desktop. Cobalt uses contextual surveys and evidence-based assessments to triage individual users to curated content and appropriate group or individual support. The current, and usual care, model is a digital version of what exists at other institutions where resources are available for staff to access wellness and mental health services. The usual care Cobalt model requires staff to personally initiate multiple steps: 1) identify a personal need for support, 2) know where to seek support and care, 3) schedule an appointment, and 4) attend the appointment. In this context, the individual has to pull the resources they need and there may be several barriers to completing each step. This is particularly relevant as mental health conditions can compromise insight, motivation, and decision making, thereby making self-directed engagement in care (pull) more challenging. The RCT will compare this pull model with a new model, the intervention named Cobalt+, which will proactively reach out to and engage individuals in order to reduce barriers in identifying a need for and accessing mental health care. The intervention group will have access to the same Cobalt resources as those in the usual care group but will also receive a comprehensive suite of services including: 1) monthly automated text messaging reminders and links to Cobalt resources 2) intermittent mental health assessments (PHQ-9, GAD-7) at enrollment, month 2, month 4 and month 6 which triage individuals to an opt-out appointment with a resilience coach, psychotherapist, or psychiatrist based on their results. Of note, all employees will have access to Cobalt whether they are in the study or not. Through the proposed RCT we will evaluate whether the intervention, a proactive model of care delivery (Cobalt+) impacts the primary outcome measure (depression and anxiety) and secondary outcome measures (well-being, satisfaction with access to care, and measures of work productivity [e.g. absenteeism]). Difficulties due to health conditions mental health domains will be assessed at the month 9 follow up survey.

9. Analysis Plan

We will conduct an intent to treat (ITT) analysis. The ITT analysis will include all randomized participants in the groups to which they were randomly assigned. Baseline demographic and clinical characteristics will be reported as frequency and percent for categorical variables and median and distribution for continuous variables. We will compare baseline characteristics between intervention and control arms using t-tests for continuous variables and chi-squared tests for categorical variables. The goal of these comparisons will be to determine if the two arms are balanced on baseline variables after randomization. Our primary analysis will focus on the difference in PHQ-9 and GAD-7 scores at 6 months between the two arms. We will estimate and test differences in means between arms using two- sample

t-tests and generalized linear models that account for baseline measurements of PHQ-9 and GAD-7. Missing data will be assessed for patterns and multiple imputation will be used if deemed appropriate. Baseline covariates that are found to be imbalanced between arms may be adjusted for in the model if they were deemed to be potential confounders a priori to adjust for potential confounding and for efficiency gain. We will use all available PHQ-9 and GAD-7 scores on eligible patients from randomization through the last observation. A p0.05 will be deemed statistically significant but emphasis will be placed on point estimates and confidence intervals. Secondary outcomes of mental healthcare utilization, satisfaction with connection to services, and qualitative feedback on mobile engagement will also be modeled using GEE with mean models specified based on the distribution of the specific outcome (e.g. Poisson or negative binomial for number of mental healthcare visits, logit for binary responses). Potential associations between system-level or regional level case burden and PHQ-9 and GAD-7 scores at baseline and over-time will be assessed with generalized linear models and GEE. Exploratory Aim 1: Statistical approach: The statistical approach for studying HTE will be to test for the statistical interaction between the intervention and race and separately by gender in the GEE models described above. Although our study was not designed specifically to detect HTE, if 20% of our trial participants are Black (based on targeted recruitment efforts), our study will achieve 80% power (assuming type I error of 0.05) to detect a 1.8 difference in mean GAD-7 scores and a 2.0 difference in mean PHQ-9 scores between arms at 6 months. We anticipate that at least 50% of participants will be female. Hence, our study is powered to detect a 1.2 difference in mean GAD-7 scores and a 1.3 difference in mean PHQ-9 scores between arms at 6 months among women.

Aim 2: Power: no power calculation will be performed for this qualitative aim consistent with prior qualitative aims we will aim to interview 80 participants from Aim 1 and evaluate themes until saturation Analysis: Using thematic analysis, we will analyze HCW interview transcripts to identify recurring themes in attitudes and beliefs toward mental health, well-being, and mental health care or resources. Interview audio will be transcribed and uploaded into Nvivo software (available as a Penn resource). In total we plan for up to 80 semi-structured interviews, or until inductive thematic saturation101 is reached in each group meaning no new themes are emerging. Analysis of qualitative, semi-structured interviews utilizes a series of systematic steps in transcribing, developing a code for thematic content, and subsequent coding of interviews. A modified grounded theory approach will be used to evaluate interview transcriptions. Analysis will begin shortly after the first few interviews are completed. An emergent, iterative open coding process will be followed and two trained coders from the research team will jointly read five transcripts and draft the outline of a codebook. Each coder will then independently code five more transcripts and adjust the codebook as needed. We will review the codes, resolve discrepancies and finalize coding procedures. Following adjudication of any areas of discrepancy, the research team coders will be assigned batches of transcripts for independent coding, sharing 25% of the transcripts in each batch for ongoing inter-rater agreement assessment. We will review coding for interrater reliability, measured by Cohens kappa (0.8), and overall comprehensiveness. If a high kappa is not achieved, further double coding and refining of the code will continue. This approach to coding and analyzing qualitative data has been widely used. Strategies used to ensure reliability and validity in the qualitative data will include a comprehensive audit trail, checks between coders, team debriefing, and corroboration of findings across participants.

10. Subject Confidentiality

The research team will exercise extreme caution with identifiable private information. Patients randomized to the intervention arm will receive text messages from Way to Health. In the patients informed consent, they are instructed and agree by signing the document that they will not disclose personal health information (PHI) on the Way to Health texting platform and only respond to the text

message prompts. When patient participants (both control and intervention) text into the Way to Health or complete a survey, the research team will receive email notification immediately. The research team is instructed to review the message within 1-4 hours of delivery, Monday through Friday, 8 am to 5pm. The line will be closed on Saturday and Sunday however patient participants will receive an out of office message if they text in; the messages notes, Thank you for reaching out, we will get back to you when we get back in the office! and a Patient Inquiry Incident is created. An exception to confidentiality is if significant suicidal ideation, is exhibited through text message or via PHQ-9 question 9 or 9 or DSM-5 question 11. PHQ-9, Question 9 asks about thoughts of being better off dead or of hurting oneself in some way over the last two weeks with an associated scale delineating not at all, several days, more than half the days, or nearly every day. The DSM-5, question 11 asks about During the past TWO (2) WEEKS, how much (or how often) have you been bothered by the following problems? Thoughts of actually hurting yourself? with an associated scale delineating None: Not at all; Slight Rare, less than a day or two; Mild: Several days; Moderate: More than half the days; and Severe: Nearly every day. Those indicating any response beyond not at all or none not at all will be triaged to Penn Behavioral Health Corporate Services / EAP. This is the current standard of care and will be the same for both Cobalt and Cobalt plus in this trial. EAP will receive only the participants phone number via email. If EAP logs into Cobalt, a HIPPA compliant platform, they can view the studys participants phone number, first and last name and PHQ-9 score and PHQ-9 question 9 score. In case Penn Behavioral Health Corporate Services / EAP is unable to contact a study participant within the 72-hour period, a research team member will call the study participant and complete the Ask Suicide-Screening Questions (ASQ) and NIMH TOOLKIT: OUTPATIENT Brief Suicide Safety Assessment.

10.1 Subject Privacy

Aim 2 transcripts and audio files will be saved on a Penn Medicine managed, HIPAA compliant server. Interview audio-recordings will be transcribed verbatim and all identifiers will be removed. The recording and transcript will be kept a secure and locked area with access limited to designated researchers. After we analyze the recordings, we will destroy recordings after data analysis or completion of the study. All encounters will take place online via BlueJeans or Way to Health, HIPAA compliant platform. or Zoom videoconference. All encounters will take place in-person in a secure private room or online via Zoom (other Penn approved video conferencing platform) or Way to Health, HIPAA compliant platform. When utilizing Zoom for audio recordings, additional steps will be made by the research team to ensure participant safety. Based on guidelines, researchers will ensure the following settings for privacy and compliance for Penn Zoom. 1) Researchers will turn off the Personal Meeting ID setting and instead opt for the meeting ID to be generated automatically, allowing for new links and meeting IDs to be created for each meeting. 2) Research staff will turn off Cloud Recordings and ensure only local recordings that are HIPAA compliant are used as they are encrypted and managed by PMACS/DART or UPHS. Interview audio-recordings will be transcribed verbatim and all identifiers will be removed. The recording and transcript will be kept a secure and locked area with access limited to designated researchers. After we analyze the recordings, we will destroy recordings after data analysis or completion of the study. All survey data will be stored in a secure, web-based database (Way to Health or REDCap) where a study ID number will be generated for each patient. A link between the study ID number and the patient PHI will need to be maintained to ensure that the study staff can track recruitment efforts to potential participants and to avoid contacting any patients who have previously declined to participate. To ensure that patient confidentiality is preserved, individual identifiers (such as name) are stored in a single password protected system that is accessible to study research, analysis and IT staff only. This system is hosted on site at UPenn and is protected by a secure firewall. Once a participant is in this system, they will be given a unique study ID number. Any datasets and computer

files that leave the firewall will be stripped of all identifiers besides the study ID and individuals will be referred to by their study ID only. The study ID will also be used on all analytical files. Way to Health and REDCap are secure web applications for building and managing online surveys and databases. Privacy of all study data will be maintained by restricting access to the identifiable information only to approved study staff who have received subject confidentiality and privacy training. Study coordinators will access patient contact information from the database to conduct recruitment phone calls. The study coordinator will review the consent script, which will include a description of the voluntary nature of participation, the study procedures, risks and potential benefits in detail. Participants will be told that all information will be kept strictly confidential, except as required by law. Subjects will be provided a copy of the consent document. All efforts will be made by study staff to ensure subject privacy. Enrollment will be conducted by the study coordinators who will enter patient information directly into the Way to Health platform once a participant has consented to participate. This database is hosted on a secure server as detailed in the subject confidentiality section. Study coordinators may have to contact patients in the intervention and their support partners during the course of the study and will use the WTH database to access contact information to facilitate this contact. If suicidal ideation, is exhibited through text message or via PHQ-9 question 9 or DSM-5 Question 11. PHQ-9, Question 9 asks about thoughts of being better off dead or of hurting oneself in some way over the last two weeks with an associated scale delineating not at all, several days, more than half the days, or nearly every day. The DSM-5, question 11 asks about During the past TWO (2) WEEKS, how much (or how often) have you been bothered by the following problems? Thoughts of actually hurting yourself? with an associated scale delineating None: Not at all; Slight Rare, less than a day or two; Mild: Several days; Moderate: More than half the days; and Severe: Nearly every day. Those indicating any response beyond not at all or none not at all will be triaged to Penn Behavioral Health Corporate Services / EAP. This is the current standard of care and will be the same for both Cobalt and Cobalt plus in this trial. In case Penn Behavioral Health Corporate Services / EAP is unable to contact a study participant within the 72-hour period, a research team member will call the study participant and complete the Ask Suicide-Screening Questions (ASQ) and NIMH TOOLKIT: OUTPATIENT Brief Suicide Safety Assessment. This will be explained to the participant in the consent process and when the details of study participation are explained by the coordinator. If a participant books an appointment at the TEAMS Clinic, the research team will receive data from their PennChart such as if appointments are fulfilled or cancelled/skipped. Survey data will be submitted to the National Institute of Mental Health Data Archive (NDA) at the National Institutes of Health (NIH). NDA is a large database where deidentified study data from many NIH studies are stored and managed. De- identified study data means that all personal information (such as name, address, birthdate and phone number) is removed and replaced with a code number. PHI will not be shared with anyone outside the parameters of the study as detailed in the Consent/HIPAA process.

10.2 Data Disclosure

Survey data will be submitted to the National Institute of Mental Health Data Archive (NDA) at the National Institutes of Health (NIH). NDA is a large database where deidentified study data from many NIH studies are stored and managed. De- identified study data means that all personal information (such as name, address, birthdate and phone number) is removed and replaced with a code number. PHI will not be shared with anyone outside the parameters of the study as detailed in the Consent/HIPAA process.

10.3 Data confidentiality

The following methods will be employed to protect patient PHI for this research study:

x Computer-based files will only be made available to personnel involved in the study through the use of access privileges and passwords.

x Prior to access to any study-related information, personnel will be required to sign statements agreeing to protect the security and confidentiality of identifiable information.

x Wherever feasible, identifiers will be removed from study-related information.

x A Certificate of Confidentiality will be obtained, because the research could place the subject at risk of criminal or civil liability or cause damage to the subject's financial standing, employability, or liability.

x Precautions are in place to ensure the data is secure by using passwords and encryption, because the research involves web-based surveys.

x Audio and/or video recordings will be transcribed and then destroyed to eliminate audible identification of subjects.

11. Consent Process Overview

Participants will enroll in this study via a remote recruitment process. The study coordinator will review the consent script, which will include a description of the voluntary nature of participation, the study procedures, risks and potential benefits in detail. Participants will be told that all information will be kept strictly confidential, except as required by law. Subjects will be provided a copy of the consent document. All efforts will be made by study staff to ensure subject privacy. Enrollment will be conducted by the study coordinators who will enter patient information directly into the Way to Health platform once a participant has consented to participate.

11.1 Potential Study Risks

We are requesting a waiver of the requirement to document consent and HIPAA authorization with a signature for participants enrolled into this study since we believe that the research presents no more than minimal risk of harm to subjects and involves no procedures for which written consent is normally required outside of the research context. [45 CFR 46.117(c)(2)] Participants will enroll in this study via a remote recruitment process, and therefore, we will read the IRB-approved Consent/HIPAA script over the phone to each participant and ask them to provide verbal consent and verbal HIPAA authorization for use of their data in the study. After a patient provides verbal consent, the coordinator will select this option on the Consent/HIPAA screen on the participants' profile that was created during enrollment on the WTH platform for this study. A copy of the Consent/HIPAA document will be included in the device packet provided to them by mail to the patients' home address.

11.2 Potential Study Benefits

All participants will have access to Cobalt, digital mental health and well-being platform which provides stepped care based on needs. Additionally, participants using either Cobalt or Cobalt+ may identify depression or anxiety otherwise unknown to them and then will be connected to care. The direct benefit to human subjects is in the use of Cobalt (usual care) and for those who are in the Cobalt+ intervention arm, access to a pushed model of care where resources are opt-out. The information

learned from this study will hopefully help contribute to the fields of digital health and mental health. The risks are reasonable in relation to the importance of knowledge expected to be gained. Information learned from this study could improve our understanding of how digital platforms can be used to engage participants, measure mental health using validated scales, and connection individuals to resources and care. We will also gain insights about how these platforms impact access to care and perceived barriers to care in healthcare workers. Ultimately, the data can be used to better understand and improve strategies for protecting and supporting the mental health of healthcare workers throughout the COVID-19 pandemic and its associated phases, but also in the future.

11.3 Alternatives to Participation

Not participate in the study. As a reminder, all Penn Medicine and University of Pennsylvania have access to Cobalt resources, regardless of their participation in the study.

11.4 Data and Safety Monitoring

The study will be single-blinded and the study biostatistician will determine when to unblind the study. Given that the study compares the same process delivered as either a pull (usual care = Cobalt) or a push (intervention = Cobalt +) we believe risk is low. Nevertheless we will engage a 3-member independent Data and Safety Monitoring Board to oversee the trial. In accordance with conventional practices, we will not identify members in advance so as not to disqualify potential proposal reviewers. Given that the study compares the same process delivered as either a pull (usual care) or a push (intervention = well-being platform) we believe risk is low. Nevertheless, we will engage a 3-member independent Data and Safety Monitoring Board to oversee the trial. In accordance with conventional practices, we will not identify members in advance so as not to disqualify potential proposal reviewers. These three external investigators will have expertise in mental health and wellness, employee health, and digital health. In accordance with conventional practices, we will not identify members in advance so as not to disqualify potential proposal reviewers. The responsibilities of the DSMB will include: Reviewing the research protocol, consent, and plans for DSMB Evaluate the progress of the trial: recruitment, retention, data quality, and adverse events Protection of participant safety Ensure high standards for privacy and no breech of data security Provide written summary reports to the PI (Merchant) and investigators after convening Provide a written summary report to the NIH funding body if required This study will not have pre-specified stopping rules. The study will be single-blinded and the study biostatistician will determine when to unblind the study. DSMB members will be independent from any professional or financial conflict of interest with the research project and/or study investigators. The DSMB will meet at least once yearly via phone or virtual conference calls for the duration of the project. The DSMB will elect a Chair to moderate the meetings. At the initial meeting, the DSMB will review and approve all study protocols before study initiation to ensure participant safety. Protocols will include formal procedures for reporting and tracking all adverse reactions to the NIH and IRBs; tracking progress in the study; and identifying any need for premature termination of the protocol. At subsequent meetings, the DSMB will be provided with summary study progress reports and adverse events. The DSMB will provide a summary report following each meeting. We will not require the DSMB to conduct interim analyses of data prior to the end of the study. We ensure that there are no conflicts of interest with the DSMB study team or proposed institutions. Per our Data Safety and Monitoring plan, submitted on 10/14/2020, pages 7-9 outlines how the research team and DSMB will handle and report unexpected and adverse events. A brief overview is below: According to the Penn Manual for Clinical Research / Office for Human Research Protections, an unanticipated problem is any incident, experience, or outcome that meets all of the following criteria: unexpected; related or possibly

related to participation in the research; and suggests that the research places subjects or others at a greater risk of harm than was previously known or recognized. Since these are, by definition, unanticipated, it is impossible to state, a priori, what kind of unanticipated problems we expect in the trial. However, unanticipated problems in the trial might include differential rates across treatment groups of increased rates of mental health appointments or emotional distress. The trial will rely on a participants family or friend to report death and a participant self-report for other unanticipated problems.

Cobalt Plus Data and Safety Monitoring Plan
14 October 2021

1. Summary of the Protocol:

A brief description of the study design (e.g., interventions, procedures, tests and scans, biospecimen collection, interviews and focus groups, study visits)

We will conduct a prospective two-armed randomized control trial to test the effect of tailored mental health support on self-reported HCW mental health (anxiety and depression) during the response and recovery phases of the COVID-19 pandemic. The control group will have access to Penn Cobalt, a digital platform for mental health care and support, which is usual care for all Penn Medicine employees. The Cobalt platform is web-based and is accessible from any device or desktop. Cobalt uses evidence-based surveys and assessments on mental health to triage individual users to curated content and appropriate group or individual support. The current, and usual care, model represents a digital version where resources are available for staff to access wellness and mental health services on their own. The usual care Cobalt model requires staff to initiate and navigate multiple steps on their own which include:

- identify a personal need for support,
- know where to seek support and care,
- schedule an appointment, and
- attend the appointment.

In this standard of care model, the individual staff member has to not only identify a need, but then "pull" the resources they need towards them. There may be several barriers to completing each step including time, stigma, lack of knowledge, among others. This is particularly relevant as mental health conditions can compromise insight, motivation, and decision making, thereby making self-directed engagement in care ("pull") more challenging.

The RCT will compare the standard "pull" model with a new model, the intervention named "Cobalt+," which will proactively reach out to and engage individuals in and attempt to reduce barriers inherent to identifying a need for and accessing mental health care. The intervention group will have access to the same Cobalt resources as those in the usual care group but will also receive a comprehensive suite of services delivered to them including:

- monthly automated text messaging engagement and links to Cobalt resources
- intermittent mental health assessments (PHQ-9, GAD-7) at enrollment, month 2, month 4 and month 6 which triage individuals to an appointment with a resilience coach, psychotherapist, or psychiatrist based on their results.

Of note, all employees will have access to Cobalt whether they are in the study or not. The proposed RCT will evaluate whether a proactive model of care delivery (the intervention named Cobalt+) impacts the primary outcome measure (depression and anxiety) and secondary outcome measures (well-being, satisfaction with access to care, and measures of work productivity [e.g. absenteeism]) as compared to usual care.

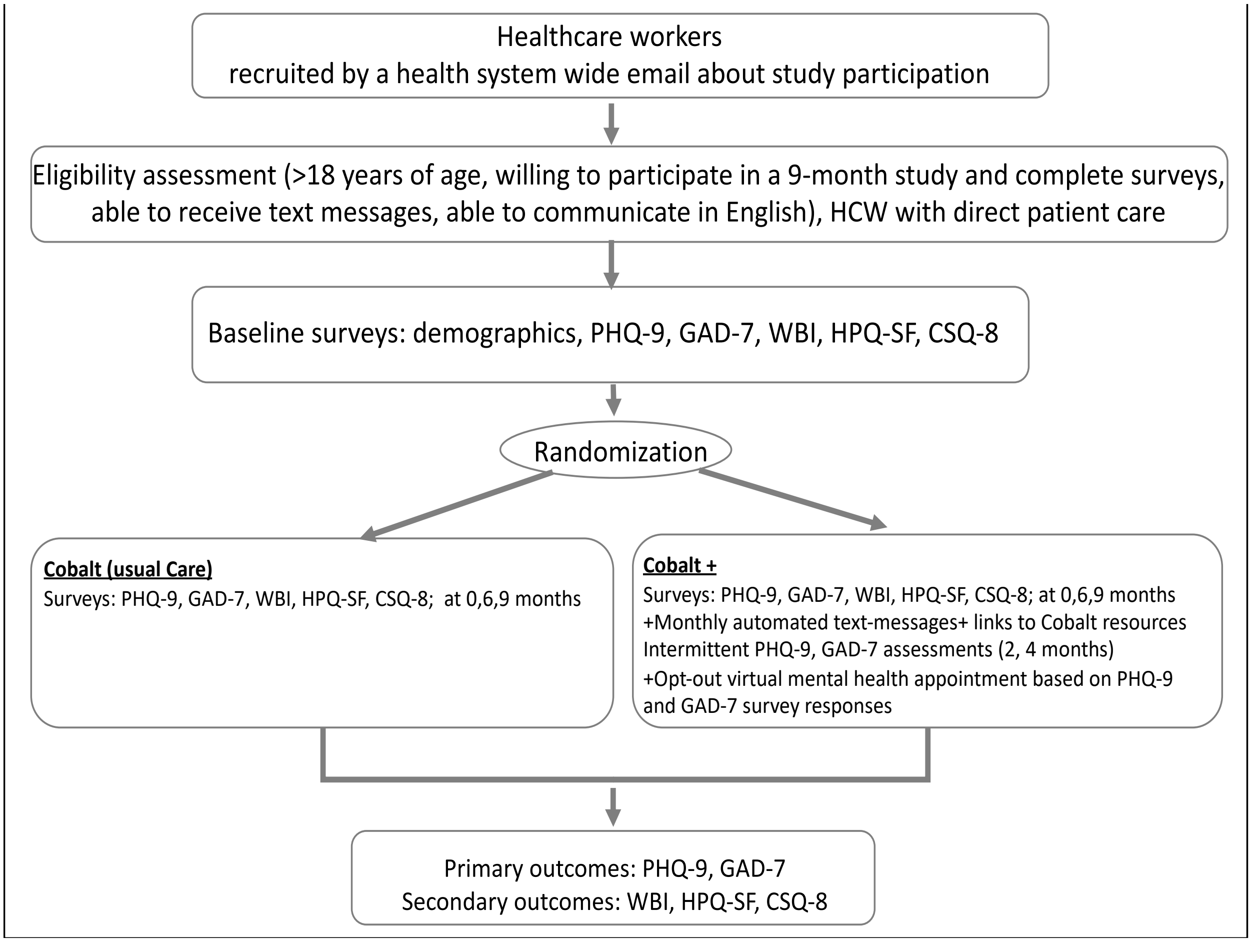

Primary and secondary outcome measures/endpoints

The primary outcome will be depression and anxiety as measured by the Patient Health Questionnaire (PHQ-9) and General Anxiety Disorder (GAD-7) respectively.

- PHQ-9 and GAD-7 have been validated and used in multiple studies and provide easy, simple scales to quickly assess for depression and anxiety. PHQ9 is a 9-item score which ranges from 0 (least depressed) to 27 (most depressed).
- GAD-7 is a 7- item scale which assess the frequency of anxiety symptoms over the past two weeks on a 4-point Likert-scale ranging from 0 (never) to 3 (nearly every day). The total score of GAD-7 ranged from 0 to 21, with increasing scores indicating more severe functional impairments as a result of anxiety.

Secondary outcomes are:

- Well-being index: The Well-being index (WBI) is a nine-question survey validated for use in HCW populations and considered important to health systems in managing the well-being of their workforce.
- Work productivity: To evaluate work productivity, we will use the World Health Organization Health and Work Performance Questionnaire (HPQ) short form. The HPQ is a validated self-report instrument designed to estimate the workplace costs of health problems with regard to reduced job performance, absence due to sickness, and work-related injuries and accidents. We will administer the short form which is an 11-question survey focused on absenteeism and presenteeism.

- Satisfaction with care: Client Satisfaction with Care (CSQ)-8 is a brief 8-item unidimensional well-established scale for assessing patient's satisfaction with overall outpatient health care. It has been shown to be reliable and valid in assessing care across a range of settings.

All potential study participants will be screened via a self-report survey on REDCap (a HIPAA compliant survey and data capture platform). Criteria will be evaluated through REDCap branching logic.

Sample size and target population
We aim to enroll 1250 participants with the anticipation that there will be 20% attrition.

We will recruit health care workers who provide direct patient care approximately 4 hours/week (on average 192 hours/ 48 weeks in a year) in the study time frame across the Penn Medicine Health System, this includes: the Hospital of the University of Pennsylvania, Penn Presbyterian Medical Center, Pennsylvania Hospital, Chester County Hospital, Penn Medicine Lancaster General Hospital and Penn Medicine Princeton. All sites will be included in one central Institutional Review Board system.

Inclusion/exclusion criteria and how the criteria will be evaluated
There are no inclusion and exclusion criteria for any subpopulation based on race/ethnicity, age, gender, status as a vulnerable population. Considering the disproportionate impact of COVID-19 on underrepresented minority populations and historic barriers to access to mental health care for this population, we will aim for inclusivity of this group in study participation. Exploratory Aim 1 specifically seeks to determine if the effect of the intervention varies across patient subgroups (e.g., gender and race).

Eligibility criteria include:

1) age 18 years or older
2) regular, daily access to a smartphone
3) able to communicate fluently in English (as the current Cobalt assessments and resources are in English at this time)
4) provide direct patient care (e.g., physicians, nurses, certified nursing assistants, lab technicians, radiology technicians, physical therapist, occupational therapist, pharmacists, pharmacy technicians, patient registration staff, receptionists/ patient intake coordinators, environmental service personnel) approximately 4 hours/week (on average 192 hours/ 48 weeks in a year) in the study time frame.

Eligible participants will be approached to complete informed consent. Any HCW meeting these criteria will be eligible to participate in the research; we will not exclude any potential participant based on a priori criteria.

Exclusion criteria include:

1). Under 18 years of age
2). does not have regular, daily access to a smartphone
3). unable to communicate fluently in English (as the current Cobalt assessments and resources are in English at this time)
4). does not provide direct patient care approximately 4 hours/week (on average 192 hours/ 48 weeks in a year) in the study time frame
5). Not willing to provide informed consent.

2. Roles and Responsibilities

Identification and description of individuals responsible for monitoring the trial (e.g., PI, ISM, DSMB), their roles, qualifications, and the frequency of the monitoring activities.

The Data and Safety Monitoring Board (DSMB) is an independent group of experts convened to protect the safety of research subjects and to ensure that the scientific goals of the project are being met. They will be an independent group of experts charged with reviewing study data for data quality and integrity, adherence to the protocol, participant safety, and study conduct and progress. During the intervention phase, the DSMB will monitor survey data (as available) and will monitor and investigate reports of safety concerns relating to study participants. The DSMB is a multidisciplinary group with a written charge, with members appointed by the PI. The DSMB can be advisory to the NIMH as needed. DSMB composition includes the following:

(1) A physician with expertise in health systems and mental health conditions

(2) A mental health epidemiologist with expertise in longitudinal analysis, field trials, and clinical trial data

(3) An epidemiologist, statistician, computer scientist, and a mental health services researcher studying the outcomes, quality of care, and pharmacoepidemiology of patients with serious mental disorders

DSMB members will be independent from any professional or financial conflict of interest with the research project and/or study investigators. The DSMB will meet at least once yearly via phone or virtual conference calls for the duration of the project. The DSMB will elect a Chair to moderate the meetings. At the initial meeting, the DSMB will review and approve all study protocols before study initiation to ensure participant safety. Protocols will include formal procedures for reporting and tracking all adverse reactions to the NIH and IRBs; tracking progress in the study; and identifying any need for premature termination of the protocol. At subsequent meetings, the DSMB will be provided with summary study progress reports and adverse events. The DSMB will provide a summary report following each meeting. We will not require the DSMB to conduct interim analyses of data prior to the end of the study. We ensure that there are no conflicts of interest with the DSMB study team or proposed institutions.

Below are anticipated DSMB responsibilities:

- Review the research protocol, informed consent documents and plans for data safety and monitoring;
- Recommend subject recruitment be initiated after receipt of a satisfactory protocol;
- Evaluate the progress of the trial, including periodic assessments of data quality and timeliness, recruitment, accrual and retention, participant risk versus benefit, and other factors that can affect study outcome;
- Consider factors external to the study when relevant information becomes available, such as scientific or therapeutic developments that may have an impact on the safety of the participants or the ethics of the trial;
- Review study performance, make recommendations and assist in the resolution of problems reported by the PI;
- Protect the safety of the study participants;
- Monitor and investigate suspected and unexpected severe adverse events (SAEs) and to unanticipated problems and determine if study is related or not;
- Report to NIH on the safety and progress of the trial;
- Make recommendations to the NIH concerning continuation, termination or other modifications of the trial based on the observed beneficial or adverse effects of the treatment under study;

- Ensure the confidentiality of the study data and the results of monitoring; and,
- Assist the NIH by commenting on any problems with study conduct, enrollment, sample size, and/or data collection;
- Provide written summary reports to the PI (Merchant) and investigators after convening;
- Provide a written summary report to the funding body if required.

We propose the following for the DSMB meetings:

- At the first meeting the DSMB will discuss the protocol, suggested modifications, and establish guidelines for study monitoring by the Board.
- The DSMB will meet at least two times annually as determined by the Chairperson.
- An emergency meeting of the DSMB may be called at any time by the Chair or by the NIH should participant safety questions or other unanticipated problems arise.
- Meetings shall be closed to the public because discussions may address confidential participant data.
- Meetings are attended by the Principal Investigators and members of their staff.
- Meetings may be convened as conference or videoconference calls as well as in-person.

3. Trial Safety

Description of any specific events that would preclude a participant from continuing the intervention

Death of a participant would constitute preclusion from continuing the intervention.

Description of the potential risks and the measures in place to protect participants against foreseeable risks

There are minimal risks to participants in this study. The potential risks to study participants include:

- Breach of confidentiality and privacy as information about each participant including their health-related information
  - To protect against this, study team will closely adhere to Penn Medicine IRB protocols and minimize the risk of breach of data and confidentiality, we will use secure, encrypted servers to host the data and conduct the analysis.
- Participants will also complete mental health surveys and the questions within these assessment tools may be sensitive or emotional for participants.
  - To mitigate risks of self-harm, all study participants will sign an informed consent explaining that all Penn Medicine employees have access to Cobalt resources regardless of their participation in the trial. Study participants will also receive guidance on 24/7 confidential hotlines and services if they experience thoughts of self-harm.
- Individuals known to the participant who view messages or survey responses on the participant's phone.
  - To protect against this, all participants will be informed of this potential risk and be educated on how to password protect their device and limit access using the device’s inherent security features.

Description of the consent/assent procedures (e.g., by whom, how and under what conditions will a subject be consented)

All study participants will independently read and sign the informed consent document on Way to Health, a HIPPA compliant platform and receive a pdf of the document after they enroll. The informed consent process informs the participant of all pertinent aspects of the study, provides contact

information for the study team should the participant have any questions, and indicates the study is voluntary and that the participant has the right to stop at any time. Participants will have access to the document at all times for their review.

Description of the mechanisms in place to protect subject privacy (e.g., interviews will take place in a private room, whether results of testing data will be shared with participant's legally authorized representative, privacy for minors, secure means of communication between investigators and participant, e.g., telephone, web portal)

Mechanisms to protect subject privacy include:

- To minimize the risk of breach of data and confidentiality, we will use secure, encrypted servers to host the data and conduct the analysis.
- All portions of the Cobalt Plus data system will be password protected using a standard challenge/response system coupled with a user-specific identity system requiring users to log in with their personal PIN and password, which are checked before the login is completed.
- Once the user is logged in, all activities are stamped with the user's PIN and date-time stamp. Knowledge of participant's and email addresses will be limited to staff who need to know that information.

Description of the trial stopping rules for the study, if any (e.g., increased suicidal ideation, greater than expected morbidity or mortality rate).

No anticipated events are applicable to stopping the trial.

Description of the plan for management of <u>incidental findings</u> (e.g., a brain tumor or potential structural abnormality discovered during a scan).

This study will not have incidental findings. All participants in control and intervention arms who complete assessments and are categorized as severe or at immediate risk for self-harm will be connected to resources and a mental health professional. No additional participant-level data will be collected and thus no other incidental findings will be captured.

Description of the process for the disclosure of any conflicts of interest that may potentially challenge participant safety or bias the data and how the conflict will be managed

Co-Investigator David Asch reported a conflict of interest, to which this has been disclosed to all study team members and noted in the informed consent as, "Please note that there are other factors to consider before agreeing to participate such as additional procedures, use of your personal information, costs, and other possible risks not discussed here. Penn Medicine Cobalt resources and delivery system used as a part of this protocol was developed by inventors at Penn Medicine and if it were to be successful, the University of Pennsylvania would benefit financially."

Description of the data security in place to protect the confidentiality of the data (e.g., password protected encrypted electronic records) and any limits to confidentiality (e.g., suicidal ideation, child abuse)

Data security practices in place to protect the confidentiality of the data include:

- All data will be stored on the Way To Health Platform, a HIPAA compliant platform to research, develop and deploy evidence-based patient engagement strategies.
    - Way to Health uses a role-based access control (RBAC) approach to assure that participant confidentially and study integrity is preserved.
    - Access and visibility is primary governed by the role of the individual accessing the system.

  - Access is granted by invitation only and can be revoked at any time. All Way to Health servers are managed by Penn Medicine Academic Compute Services (PMACS).
  - All data at-rest is stored on encrypted disks using encryption keys managed by Way to Health. Encrypted disks use AES encryption with a minimum of 256-bit keys, or keys and ciphers of equivalent or higher cryptographic strength.
  - User passwords are never stored in clear text; they are salted and hashed to eliminate data leakage. All data transmission is encrypted end to end using encryption keys managed by Way to Health.
  - Transmission encryption keys use a minimum of 2048-bit RSA keys, or keys and ciphers of equivalent or higher cryptographic strength (e.g., 256-bit AES session keys in the case of IPsec encryption).
  - Data downloads are generally prohibited by policy.
  - Where appropriate, most datasets are blinded of all personally identifiable information when exported for analysis. A limited number of exports including identifiers exist to assist research staff with recruitment tracking and study management efforts.
  - These datasets are only accessible to certain user roles. These user roles are required to sign and adhere to a W2H Security Agreement as described above.
  - To monitor ongoing usage of the system and identify unauthorized usage of the system, all access to the application and the database are logged automatically.
- All participants will be informed that the information they provide will be held in confidence to the extent that the law allows, but that the exception to this confidentiality is any disclosure of potential for immediate harm of themselves or others, such as active suicidal or homicidal ideation or child abuse. The participants will be notified prior to participation that if any of these issues are raised, the researchers will take whatever steps are necessary to protect the subject or others, including bringing risk of harm to the attention of the proper authorities. This applies only to direct verbal or written disclosure by the patient.

4. Reportable Events

Description of the process and timelines (e.g., hours, days) for collecting and reporting Adverse Events (AEs), Serious Adverse Events (SAEs), and Unanticipated Problems Involving Risks to Subjects or Others to appropriate monitoring and regulatory entities (See NIMH Reportable Events Policy for definitions and timeframes)Collection of Serious Adverse Events and Unanticipated Problems

According to the Penn Manual for Clinical Research, and as defined by the NIMH, serious adverse events (SAE) are defined by one of the following:

- Death
- Life-threatening experience
- Inpatient hospitalization or prolongation of hospitalization
- Persistent or significant disability/incapacity
- Congenital anomaly/birth defect in the subject's offspring

In this trial, the following event is considered a SAE: death of a study participant.

According to the Penn Manual for Clinical Research, adverse events (AE) are events that:

- Are untoward or unfavorable medical occurrence in a human subject; and
- Temporally associated with the subject’s participation in the research, whether or not considered related to the subject’s participation in the research

In this trial, the following events are considered an AE:

- Information that indicates a change to the risks or potential benefits of the research, in terms of severity or frequency.
- Breach of confidentiality.
- Incarceration of a participant when the research was not previously approved under Subpart C and the investigator believes it is in the best interest of the subject to remain in the study.
- Events that require prompt reporting to the sponsor.
- Complaint of a participant when the complaint indicates unexpected risks or the complaint cannot be resolved by the research team.
- Protocol violation (meaning an accidental or unintentional change to the IRB approved protocol) that placed one or more participants at increased risk, or has the potential to occur again.
- Sponsor-imposed suspension

According to the Penn Manual for Clinical Research / Office for Human Research Protections, an unanticipated problem is any incident, experience, or outcome that meets all of the following criteria:

- unexpected;
- related or possibly related to participation in the research; and
- suggests that the research places subjects or others at a greater risk of harm than was previously known or recognized

Since these are, by definition, unanticipated, it is impossible to state, a priori, what kind of unanticipated problems we expect in the trial. However, unanticipated problems in the trial might include differential rates across treatment groups of increased rates of mental health appointments or emotional distress. The trial will rely on a participant's family or friend to report death and a participant self-report for other unanticipated problems.

Specific plan and timeframe for reporting IRB and/or ISM/DSMB actions to the NIMH (e.g., protocol violations, non-compliance, suspensions, terminations)
We will use the following procedures in the event of an AE, SAE, and protocol violation. In any of these instances, the research team will alert the PI and site PIs immediately (same business day). Subsequently, the PI and site PIs will submit a report within 10 working days (with one exception) of events that meet the definition of an unanticipated problem involving risks to subjects or others. The exception is if the SAE involved death and indicates that participants or others are at increased risk of harm, in which case the PI will be required to submit a report to the IRB within three days. Reports are submitted electronically through IRB's electronic submission system. The IRB staff (in conjunction with the IRB chair when necessary) will review reports and decide whether the event meets the definition of an unanticipated problem increasing risks to subjects or others. Events that meet these criteria will be considered unanticipated problems involving risks to participants or others, will be reviewed by the convened IRB, and will be reported accordingly.

See below for a flowchart of how the IRB handles these types of violations

Relatedness	Expectedness	Reportable to IRB?	When to Report
Unrelated or Unlikely related	Expected and Unexpected	NO	N/A
Possibly, Probably, or Definitely related	Expected	NO	N/A
Possibly related	Unexpected	YES* ONLY IF: The event suggests that the research places **subjects or others** at greater risk than was previously known or recognized (i.e., changes to the study conduct are required to mitigate risk and/or participants' willingness to participate may be adversely impacted)	EXPEDITED REPORTING WITHIN 10 bus. days<br>Summarize at continuing review
Probably or Definitely related	Unexpected	YES*	EXPEDITED REPORTING WITHIN 10 bus. days<br>Summarize at continuing review
Probably or Definitely related death	Unexpected	YES*	EXPEDITED REPORTING WITHIN 3 calendar days<br>Summarize at continuing review

*Includes serious and non-serious events.

5. Data Management, Analysis, and Quality Assurance:

Identification of data sources (e.g., questionnaires, medical records, biospecimen collections, audio/video recordings)

In this study, we will:

- utilize self-reported surveys and audio recordings as our data sources.
- collect data from the Cobalt website for fidelity metrics including frequency of hyperlink use, text messages receiving a response, and participants request to stop receiving texts.
  - For the appointments, we will assess how often appointments are fulfilled or cancelled.

Description of the security measures in place to protect data sources including how the data will be labeled and stored

The trial's security measures are below:

- All study participants will provide written informed consent to their participation and will be assigned an ID number by which their data will be identified.
- The research team will exercise extreme caution with identifiable private information. Patients randomized to the intervention arm will receive text messages from Way to Health.
- All participants are instructed and agree by signing the document that they will not disclose personal health information (PHI) on the Way to Health texting platform and only respond to the text message prompts.
- All PHI will be stored on Way to Health, a HIPAA compliant research platform stored on Penn Medicine Academic Computing Services (PMACS) will be the hub for the hardware and database infrastructure that will support the project.
  - PMACS requires all users of data or applications on PMACS servers to complete a PMACS-hosted cybersecurity awareness course annually, which stresses federal data security policies under data use agreements with the university. Curriculum includes HIPAA training and covers secure data transfer, passwords, computer security habits and knowledge of what constitutes misuse or inappropriate use of the server.
- Password protection is used at the server and web portal levels for all transactions that allow entry and editing of data or provide access to sensitive subject data or administrative privileges.

    - Additionally, all PHI data will be encrypted and linked to survey data through a study ID number, with linkage and de-encryption keys activated only by a user password for which a member of the research team has been given permission to access these sensitive data (Principal Investigator Dr. Merchant and project staff who are under the direct supervision of the PI).
- The database management on these servers is built with multiple layers of security and follows best practices for securing sensitive data. The main levels of security are fourfold and include machine physical security at the IT facility at which the servers are housed, physical server security in the highly restricted server containment room, electronic server security (firewalls, passwords, encryption) restricting access to the machine, and directory access controls restricting access to these particular data.

Quality assurance measures for subject recruitment, enrollment, enrollment targets, and for the validity and integrity of the data.

The study team has undergone trainings on Good Clinical Practice guidance to ensure that the rights, safety, and well-being of study subjects are protected and consistent with the principles that have their origin in the Declaration of Helsinki. Study participants will complete self-report surveys while usage meta-data will be extracted from the Cobalt platform.

PROTOCOL CHANGES LOG

Summary of Amendments and Modifications:

Our IRB protocol included two components, Aim 1: randomized controlled trial and Aim 2: qualitative interviews. Aims 1 and 2 were conducted from 2021 – 2023. There were no major amendments to RCT study design. In May 2022, we increased the study sample from 1,250 to 1,275 to account for staff turnover and attrition rates. Most protocol modifications involved minor changes to survey instruments and consent procedures; adding and deleting key personnel to the study team.

File Name	Date of Submission	Approval date	Summary	Rationale
initial-sub-1	4/12/2021	5/12/2021	Original IRB protocol submission	Initial trial protocol submission
Mod-2	10/14/2021	11/23/2021	informed consent	updated informed consent
			grant application	submitted NIH grant application
			Data Safety and Monitoring Board plan	submitted Data Safety and Monitoring Board plan
			Recruitment materials	Updated recruitment letter
			Surveys	Modified study surveys
Mod-3	12/9/2021	12/13/2021	informed consent	updated informed consent
			text message intervention	updated text message intervention
			Data Safety and Monitoring Board plan	submitted Data Safety and Monitoring Board plan
			Recruitment materials	Updated recruitment letters, flyers, scripts, and infographics
			Surveys	Modified screening survey and study surveys
Mod-4	1/10/2022	…	informed consent	updated informed consent
			Recruitment materials	Updated recruitment letter
			Surveys	Modified screening survey and study surveys
			Safety procedures	Provided additional scripts and tools for self-harm safety assessments
Mod-5	3/3/2022	3/28/2022	Revised informed consent document	Received feedback to strengthen informed consent document
			Revised HSERA application	Received feedback to clarify HSERA protocol

Mod-6	4/4/2022	4/12/2022	Revised outreach materials	Revised text message answer choices, revised outreach and welcome email
			Edited compensation material	Migrated to Virtual Visa ClinCards
			Edited month 6 survey	Included multiple answer choices
Mod-7	4/22/2022	5/3/2022	Revised outreach materials	Edited welcome email
			HSERA protocol	Receive de-identified demographic information from Penn HR
			Edited month 6 survey	Added clarifying question
Mod-8	5/31/2022	6/6/2022	HSERA protocol	Increased attrition from 25% to 27.5%
			Edited month 6 survey	Edited question
Mod-9	7/11/2022	7/13/2022	HSERA protocol	Updated study personnel
			Edited month 6 survey	Simplified Cobalt use questions
Mod-10	11/7/2022	12/8/2022	Last text message in Aim 1	Included safety resources in last study text message to all participants
			Edited month 9 survey	Simplified and revised follow up question about Cobalt use; added question about clinical hours
			Updated Aim 2: qualitative interview guide	Updated questions
			Updated Aim 2: qualitative interview informed consent	Updated informed consent to mirror Aim 1
			HSERA protocol	Updated design to include new data storage, Aim 2 coding schema
Mod-11	1/3/2023	1/13/2023	Updated personnel	Added Sarah Beck and Rachel Howell as study personnel
Mod-12	2/20/2023	3/17/2023	Continuing Review Application	Submitted Continuing Review Application to the IRB
Mod-13	3/18/2023	4/4/2023	Updated Aim 2; Updated personnel	Added study personnel; provided clarification on Aim 2 sampling methods

Original Statistical Analysis Plan

We will conduct an intent to treat (ITT) analysis. The ITT analysis will include all randomized participants in the groups to which they were randomly assigned. Baseline demographic and clinical characteristics will be reported as frequency and percent for categorical variables and median and distribution for continuous variables. We will compare baseline characteristics between intervention and control arms using t-tests for continuous variables and chi-squared tests for categorical variables. The goal of these comparisons will be to determine if the two arms are balanced on baseline variables after randomization. Our primary analysis will focus on the difference in PHQ-9 and GAD-7 scores at 6 months between the two arms. We will estimate and test differences in means between arms using two- sample t-tests and generalized linear models that account for baseline measurements of PHQ-9 and GAD-7. Missing data will be assessed for patterns and multiple imputation will be used if deemed appropriate. Baseline covariates that are found to be imbalanced between arms may be adjusted for in the model if they were deemed to be potential confounders a priori to adjust for potential confounding and for efficiency gain. We will use all available PHQ-9 and GAD-7 scores on eligible patients from randomization through the last observation. A p0.05 will be deemed statistically significant but emphasis will be placed on point estimates and confidence intervals.

Secondary outcomes of mental healthcare utilization, satisfaction with connection to services, and qualitative feedback on mobile engagement will also be modeled using GEE with mean models specified based on the distribution of the specific outcome (e.g. Poisson or negative binomial for number of mental healthcare visits, logit for binary responses). Potential associations between system-level or regional level case burden and PHQ-9 and GAD-7 scores at baseline and over-time will be assessed with generalized linear models and GEE.

Exploratory Aim 1: Statistical approach: The statistical approach for studying HTE will be to test for the statistical interaction between the intervention and race and separately by gender in the GEE models described above. Although our study was not designed specifically to detect HTE, if 20% of our trial participants are Black (based on targeted recruitment efforts), our study will achieve 80% power (assuming type I error of 0.05) to detect a 1.8 difference in mean GAD-7 scores and a 2.0 difference in mean PHQ-9 scores between arms at 6 months. We anticipate that at least 50% of participants will be female. Hence, our study is powered to detect a 1.2 difference in mean GAD-7 scores and a 1.3 difference in mean PHQ-9 scores between arms at 6 months among women.

Aim 2: Power: no power calculation will be performed for this qualitative aim consistent with prior qualitative aims we will aim to interview 80 participants from Aim 1 and evaluate themes until saturation Analysis: Using thematic analysis, we will analyze HCW interview transcripts to identify recurring themes in attitudes and beliefs toward mental health, well-being, and mental health care or resources. Interview audio will be transcribed and uploaded into NVivo software (available as a Penn resource). In total we plan for up to 80 semi-structured interviews, or until inductive thematic saturation is reached in each group meaning no new themes are emerging. Analysis of qualitative, semi-structured interviews utilizes a series of systematic steps in transcribing, developing a code for thematic content, and subsequent coding of interviews. A modified grounded theory approach will be used to evaluate interview transcriptions. Analysis will begin shortly after the first few interviews are completed. An emergent, iterative open coding process will be followed and two trained coders from the research team will jointly read five transcripts and draft the outline of a codebook. Each coder will then independently code five more transcripts and adjust the codebook as needed. We will review the codes,

resolve discrepancies and finalize coding procedures. Following adjudication of any areas of discrepancy, the research team coders will be assigned batches of transcripts for independent coding, sharing 25% of the transcripts in each batch for ongoing inter-rater agreement assessment. We will review coding for interrater reliability, measured by Cohens kappa (0.8), and overall comprehensiveness. If a high kappa is not achieved, further double coding and refining of the code will continue. This approach to coding and analyzing qualitative data has been widely used. Strategies used to ensure reliability and validity in the qualitative data will include a comprehensive audit trail, checks between coders, team debriefing, and corroboration of findings across participants.

Amendments to the Statistical Analysis Plan

All amendments to the original SAP were made prior to unblinding of the data analyst and prior to access to outcome data. We omitted satisfaction to access to mental health services as a secondary outcome analysis since it was underpowered to detect changes over time.

Final Statistical Analysis Plan

We will conduct an intent to treat (ITT) analysis. The ITT analysis will include all randomized participants in the groups to which they were randomly assigned. Baseline demographic and clinical characteristics will be reported as frequency and percent for categorical variables and median and distribution for continuous variables. We will compare baseline characteristics between intervention and control arms using t-tests for continuous variables and chi-squared tests for categorical variables. The goal of these comparisons will be to determine if the two arms are balanced on baseline variables after randomization. Our primary analysis will focus on the difference in PHQ-9 and GAD-7 scores at 6 months between the two arms. We will estimate and test differences in means between arms using two- sample t-tests and generalized linear models that account for baseline measurements of PHQ-9 and GAD-7. Missing data will be assessed for patterns and multiple imputation will be used if deemed appropriate. Baseline covariates that are found to be imbalanced between arms may be adjusted for in the model if they were deemed to be potential confounders a priori to adjust for potential confounding and for efficiency gain. We will use all available PHQ-9 and GAD-7 scores on eligible patients from randomization through the last observation. A p0.05 will be deemed statistically significant but emphasis will be placed on point estimates and confidence intervals.

Secondary outcomes of mental healthcare utilization, satisfaction with connection to services, and qualitative feedback on mobile engagement will also be modeled using GEE with mean models specified based on the distribution of the specific outcome (e.g. Poisson or negative binomial for number of mental healthcare visits, logit for binary responses). Potential associations between system-level or regional level case burden and PHQ-9 and GAD-7 scores at baseline and over-time will be assessed with generalized linear models and GEE.

Exploratory Aim 1: Statistical approach: The statistical approach for studying HTE will be to test for the statistical interaction between the intervention and race and separately by gender in the GEE models described above. Although our study was not designed specifically to detect HTE, if 20% of our trial participants are Black (based on targeted recruitment efforts), our study will achieve 80% power (assuming type I error of 0.05) to detect a 1.8 difference in mean GAD-7 scores and a 2.0 difference in mean PHQ-9 scores between arms at 6 months. We anticipate that at least 50% of participants will be female. Hence, our study is powered to detect a 1.2 difference in mean GAD-7 scores and a 1.3 difference in mean PHQ-9 scores between arms at 6 months among women.

Aim 2: Power: no power calculation will be performed for this qualitative aim consistent with prior qualitative aims we will aim to interview 80 participants from Aim 1 and evaluate themes until saturation Analysis: Using thematic analysis, we will analyze HCW interview transcripts to identify recurring themes in attitudes and beliefs toward mental health, well-being, and mental health care or resources. Interview audio will be transcribed and uploaded into NVivo software (available as a Penn resource). In total we plan for up to 80 semi-structured interviews, or until inductive thematic saturation is reached in each group meaning no new themes are emerging. Analysis of qualitative, semi-structured interviews utilizes a series of systematic steps in transcribing, developing a code for thematic content, and subsequent coding of interviews. A modified grounded theory approach will be used to evaluate interview transcriptions. Analysis will begin shortly after the first few interviews are completed. An emergent, iterative open coding process will be followed and two trained coders from the research team will jointly read five transcripts and draft the outline of a codebook. Each coder will then independently code five more transcripts and adjust the codebook as needed. We will review the codes,

resolve discrepancies and finalize coding procedures. Following adjudication of any areas of discrepancy, the research team coders will be assigned batches of transcripts for independent coding, sharing 25% of the transcripts in each batch for ongoing inter-rater agreement assessment. We will review coding for interrater reliability, measured by Cohens kappa (0.8), and overall comprehensiveness. If a high kappa is not achieved, further double coding and refining of the code will continue. This approach to coding and analyzing qualitative data has been widely used. Strategies used to ensure reliability and validity in the qualitative data will include a comprehensive audit trail, checks between coders, team debriefing, and corroboration of findings across participants.
