# Supplementary material for: Digital Engagement Strategy and Health Care Worker Mental Health: A Randomized Clinical Trial
Source: JAMA Netw Open. 2024 May 24;7(5):e2410994. doi: 10.1001/jamanetworkopen.2024.10994 (PMC11127125; doi:10.1001/jamanetworkopen.2024.10994)
Supplement: Supplement 2. — eFigure 1. Monthly Automated Text Messages Associated With Mental Health and Penn Cobalt Resources And Mental Health Assessments (e.g., PHQ-9 and GAD-7) Appointment Triage Text Messages and Roles eFigure 2. Mental Health Assessments (e.g., PHQ-9 and GAD-7) Appointment Triage Messaging eFigure 3. Safety and Suicidality eFigure 4. Safety and Suicidality Findings eTable 1. GLM Model on Complete Cases: 6-Month GAD-7 eTable 2. Anxiety and Depression Comparison With Imputed Data eTable 3. Well-Being Index Comparison With Observed Data eTable 4. WHO-5 Comparison With Observed Data eTable 5. Leaps Comparison With Observed Data eTable 6. GEE With AR(1) [file jamanetwopen-e2410994-s002.pdf]

# Supplemental Online Content

Agarwal AK, Southwick L, Gonzales RE, et al. Digital engagement strategy and health care worker mental health: a randomized clinical trial. *JAMA Netw Open*. 2024;7(5):e2410994. doi:10.1001/jamanetworkopen.2024.10994

**eFigure 1.** Monthly Automated Text Messages Associated With Mental Health and Penn Cobalt Resources And Mental Health Assessments (e.g., PHQ-9 and GAD-7) Appointment Triage Text Messages and Roles
**eFigure 2.** Mental Health Assessments (e.g., PHQ-9 and GAD-7) Appointment Triage Messaging
**eFigure 3.** Safety and Suicidality
**eFigure 4.** Safety and Suicidality Findings
**eTable 1.** GLM Model on Complete Cases: 6-Month GAD-7
**eTable 2.** Anxiety and Depression Comparison With Imputed Data
**eTable 3.** Well-Being Index Comparison With Observed Data
**eTable 4.** WHO-5 Comparison With Observed Data
**eTable 5.** Leaps Comparison With Observed Data
**eTable 6.** GEE With AR(1)

This supplemental material has been provided by the authors to give readers additional information about their work.

© 2024 Agarwal AK et al. *JAMA Network Open.*

Study Protocol – Attached Separately

**eFigure 1. Monthly automated text messages related to mental health and *Penn Cobalt* resources and mental health assessments (e.g., PHQ-9 and GAD-7) appointment triage text messages.**

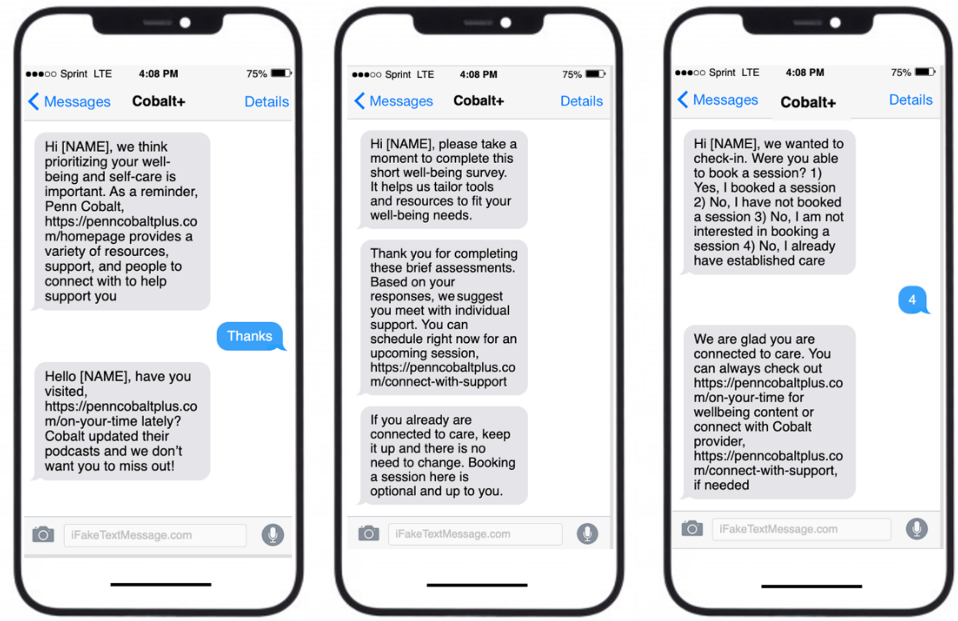


**eFigure 2. Mental health assessments (e.g., PHQ-9 and GAD-7) appointment triage messaging**

<table>
<tr><th></th><th>Mild<br>PHQ9<10 AND GAD7 < 10</th><th>Moderate<br>PHQ9>10 and <19<br>AND GAD7 >10 and <19</th><th>Severe<br>PHQ9 >19 OR GAD7 >19</th><th>SI<br>PHQ9, Q9 > 0</th></tr>
<tr><td></td><td>• Thank you for completing these brief assessments. Your responses suggest that meeting with 1:1 support could be helpful. There are sessions open today and later this week, URL<br><br>• If you already are connected to care, keep it up and there is no need to change. Booking a session here is optional and up to you.</td><td>• Thank you for completing these brief assessments. Based on your responses, we suggest you meet with individual support. You can schedule right now for an upcoming session, URL<br><br>• If you already are connected to care, keep it up and there is no need to change. Booking a session here is optional and up to you.</td><td>• Thank you for completing the brief assessments. Based on your responses, you need individual support. You can schedule right now for an upcoming session, URL. Please remember to sign into Cobalt to see their offerings.<br><br>• If you are in crisis or having thoughts of suicide or feel unsafe, reply with "A" for 24/7 confidential and free crisis services.<br><br>• [If A] Please go to your nearest emergency room or visit one of these 24/7 confidential and free services: • Penn Crisis Response Center: call 215-829-5433<br>• National Suicide Prevention Line: call 988<br>• Crisis Text Line: text HOME to 741 741 or https://www.crisistextline.org</td><td>• Hello PARTICIPANT_FIRSTNAME, thank you for completing these brief assessments. Based on your responses, you will receive a phone call from the Penn Behavioral Health Services team within 1 business day.<br><br>• Please remember, this number is not a crisis response line. If you in crisis or are having thoughts of suicide or feel unsafe, please go to your nearest emergency room or visit one of these 24/7 confidential and free services: • Penn Crisis Response Center: call 215-829-5433<br>• National Suicide Prevention Line: call 988<br>• Crisis Text Line: text HOME to 741 741 or https://www.crisistextline.org/</td></tr>
<tr><td>Two Days Later</td><td colspan="3">Hi [NAME], we wanted to check-in. Were you able to book a session?<br>A. Yes, I booked a session<br>• [If A] Glad to hear<br>B. No, I have not booked a session<br>• [If B] You can schedule right now for an upcoming appointment, URL. Please remember to sign into Cobalt to see their offerings. Need help booking an appointment? If yes, text Y.<br>C. No, I am not interested in booking a session<br>• [If C] Not a problem. Could you tell us why not?<br>D. No, I already have established care<br>• [If D] We are glad you are connected to care. You can always check out URL for wellbeing content or connect with Cobalt provider, URL, if needed. Please remember to sign into Cobalt to see their offerings.</td><td>Hi [NAME], we wanted to check in. Were you able to speak to someone from the Penn Behavioral Health Services team / EAP?<br>A. Yes<br>B. No</td></tr>
</table>

© 2024 Agarwal AK et al. *JAMA Network Open.*

## eFigure 3. Safety and Suicidality

**Participant Facing**

Participant expresses SI and immediately receives resources

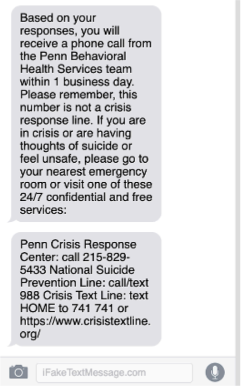


<u>After 72 hours</u>, all participants receive a follow-up text message and tailored resources based on their response

Answers **Yes** | Answers **No** | No Response

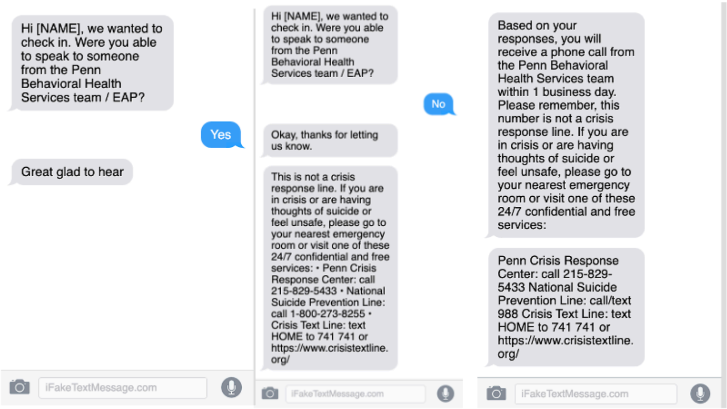


**Employee Assistance Provider (EAP) Internal Workflow**

1). EAP receives email alert

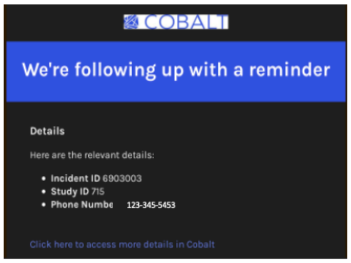


2). EAP providers logs into Penn Cobalt platform for user information to conduct safety assessment and records action taken

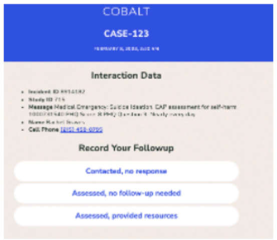


If user is "Contacted, no response" EAP receives email alert until user is contacted.

At most 3 emails in 72 hours

© 2024 Agarwal AK et al. *JAMA Network Open*.

**eFigure4. Safety and Suicidality findings**

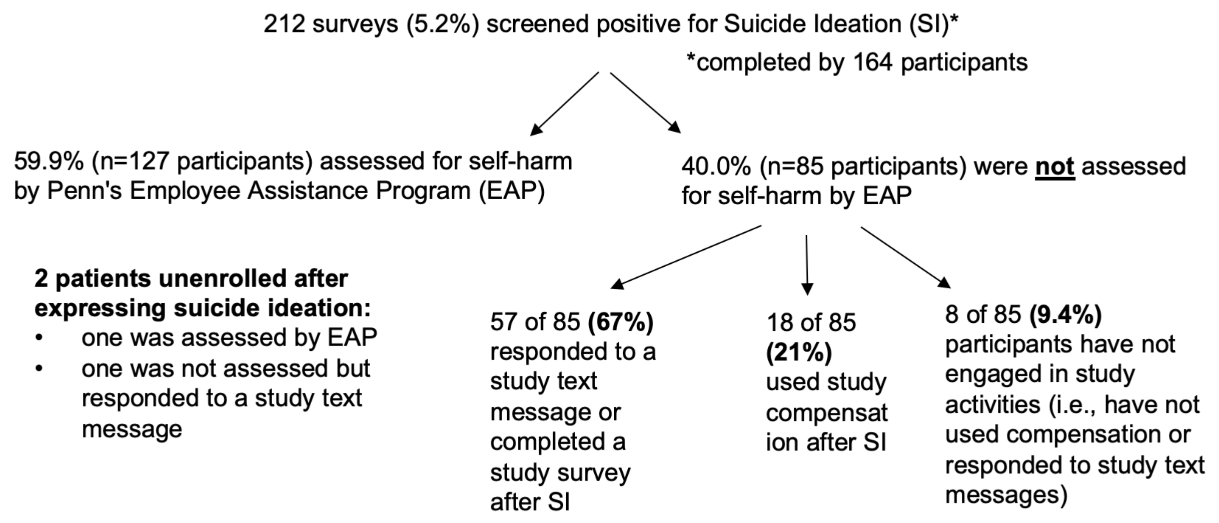


© 2024 Agarwal AK et al. *JAMA Network Open.*

**eTable 1. GLM model on complete cases: 6-month GAD-7**

| **Variables** | **β (95% CI)** | **P value** |
|---|---|---|
| Arm assignment | -0.67 (-1.13, -0.16) | 0.01 |
| Baseline GAD-7 score | 0.60 (0.55, 0.65) | <.0001 |
| Age | -0.01 (-0.03, 0.01) | 0.29 |
| Female | 0.44 (-0.24, 1.13) | 0.20 |
| Race-Ethnicity | | |
| Asian | -0.34 (-1.24, 0.55) | 0.45 |
| Black | 0.17 (-0.41, 0.76) | 0.56 |
| Other | -0.06 (-1.38, 1.25) | 0.92 |
| White | 0 | . |
| Hispanic | 0.48 (-0.64, 1.60) | 0.40 |
| Married or with partner | 0.08 (-0.43, 0.59) | 0.76 |

Participant Roles

| **Role** | **Control (%)** | **Intervention (%)** | **Total (%)** |
|---|---|---|---|
| Nurses | 210 (50) | 206 (50) | 416 (33) |
| Physicians & Advance Practice Providers | 85 (49) | 89 (51) | 174 (14) |
| Technicians (lab, pharmacy, radiology) | 71 (47) | 80 (53) | 151 (12) |
| Registration Staff | 54 (44) | 68 (56) | 122 (10) |
| Hospital and Outpatient Support (unit clerk, patient coordinator) | 58 (50) | 58 (50 | 116 (9) |
| Clinical Research | 38 (47) | 43 (53) | 81 (6) |
| Food and Environmental Services | 33 (55) | 27 (45) | 60 (5) |
| Medical Assistants | 22 (51) | 21 (49) | 43 (3) |
| Therapists (occupational, physical, speech, respiratory, nutritionist) | 23 (52) | 21 (48) | 44 (3) |
| Social Work and Behavioral Health | 13 (43) | 17 (57) | 30 (2) |
| Pharmacists | 26 (68) | 12 (32) | 38 (3) |
| **Total** | **633** | **644** | **1,275** |

© 2024 Agarwal AK et al. *JAMA Network Open.*

eTable 2. Anxiety and Depression Comparison with Imputed Data

**Paired t-test & arm comparison for GAD7, using imputed data**

| | Control | Intervention | Total | Difference | P Value |
|---|---|---|---|---|---|
| # of participants | 633 | 642 | 1275 | | |
| GAD7_bl, mean (std) | 5.79 (4.77) | 6.03 (4.88) | 5.91 (4.83) | 0.24 (-0.29,0.77) | 0.3723 |
| 6-month | | | | | |
| GAD7_6m, mean (std) | 5.57 (5.34) | 5.07 (4.97) | 5.32 (5.40) | -0.50 (-1.04, 0.04) | 0.0686 |
| 6-month change, mean (std) | -0.22 (4.71) | -0.96 (4.65) | -0.59 (4.96) | -0.74 (-1.23, -0.26) | 0.0027 |
| 6-month change, 95% CI | (-0.59, 0.15) | (-1.32, -0.60) | (-0.87, -0.32) | | |
| Paired t-test P-value | 0.2418 | <.0001 | <.0001 | | |
| 9-month | | | | | |
| GAD7_9m, mean (std) | 4.95 (4.81) | 4.32 (4.74) | 4.63 (4.69) | -0.62 (-1.16, -0.08) | 0.0237 |
| 9-month change, mean (std) | -0.85 (4.71) | -1.71 (4.48) | -1.28 (4.51) | -0.86 (-1.38, -0.34) | 0.0012 |
| 9-month change, 95% CI | (-1.21, -0.48) | (-2.06, -1.36) | (-1.53, -1.03) | | |
| Paired t-test P-value | <.0001 | <.0001 | <.0001 | | |

**Paired t-test & arm comparison for PHQ9, using imputed data**

| | Control | Intervention | Total | Difference | P Value |
|---|---|---|---|---|---|
| # of participants | 633 | 642 | 1275 | | |
| PHQ9_bl, mean (std) | 5.71 (4.74) | 5.92 (5.19) | 5.81 (4.97) | 0.21 (-0.34, 0.76) | 0.4514 |
| 6-month | | | | | |
| PHQ9_6m, mean (std) | 5.40 (5.36) | 4.81 (5.56) | 5.10 (5.22) | -0.60 (-1.23, 0.04) | 0.0649 |
| 6-month change, mean (std) | -0.31 (4.84) | -1.11 (5.54) | -0.71 (4.96) | -0.81 (-1.41, -0.20) | 0.01 |
| 6-month change, 95% CI | (-0.69, 0.07) | (-1.55, -0.68) | (-0.99, -0.44) | | |
| Paired t-test P-value | 0.1137 | <.0001 | <.0001 | | |
| 9-month | | | | | |
| PHQ9_9m, mean (std) | 5.02 (4.88) | 4.28 (5.04) | 4.64 (4.91) | -0.74(-1.29, -0.19) | 0.0086 |
| 9-month change, mean (std) | -0.69 (4.42) | -1.64 (4.77) | -1.17 (4.55) | -0.95(-1.46, -0.44) | 0.0003 |
| 9-month change, 95% CI | (-1.04, -0.35) | (-2.01, -1.27) | (-1.42, -0.92) | | |
| Paired t-test P-value | <.0001 | <.0001 | <.0001 | | |

© 2024 Agarwal AK et al. *JAMA Network Open.*

eTable 3: Well Being Index Comparison with Observed Data

| | Control | Intervention | Total | Difference | P Value |
|---|---|---|---|---|---|
| # of participants | 633 | 642 | 1275 | | |
| WBI9_bl, mean (std) | 2.70 (2.33) | 2.72 (2.23) | 2.71(2.28) | 0.02 (-0.23, 0.27) | 0.896 |
| 6-month | | | | | |
| # of non-missing records | 528 | 484 | 1012 | | |
| WBI9_bl, mean (std) | 2.70 (2.32) | 2.64 (2.22) | 2.67 (2.27) | -0.06 (-0.34, 0.22) | 0.6815 |
| WBI9_6m, mean (std) | 2.24 (2.30) | 2.05 (2.34) | 2.15 (2.32) | -0.19 (-0.48, 0.10) | 0.1912 |
| 6-month change, mean (std) | -0.46 (2.14) | -0.59 (2.20) | -0.52 (2.17) | -0.13 (-0.40, 0.14) | 0.332 |
| 6-month change, 95% CI | (-0.64, -0.27) | (-0.79, -0.39) | (-0.65, -0.39) | | |
| Paired t-test P-value | <.0001 | <.0001 | <.0001 | | |
| 9-month | | | | | |
| # of non-missing records | 498 | 473 | 971 | | |
| WBI9_bl, mean (std) | 2.66 (2.34) | 2.66 (2.25) | 2.66 (2.29) | 0.00 (-0.29, 0.29) | 0.9956 |
| WBI9_9m, mean (std) | 2.14 (2.26) | 1.72 (2.34) | 1.94 (2.30) | -0.42 (-0.71, -0.13) | 0.0043 |
| 9-month change, mean (std) | -0.52 (2.15) | -0.95 (2.43) | -0.73 (2.30) | -0.42 (-0.71, -0.13) | 0.0043 |
| 9-month change, 95% CI | (-0.71, -0.33) | (-1.16, -0.73) | (-0.87, -0.58) | | |
| Paired t-test P-value | <.0001 | <.0001 | <.0001 | | |

eTable4: WHO-5 Comparison with Observed Data

| | Control | Intervention | Total | Difference | P Value |
|---|---|---|---|---|---|
| # of participants | 633 | 642 | 1275 | | |
| WHO5_bl, mean (std) | 13.13 (5.33) | 13.10 (5.26) | 13.12 (5.29) | -0.03 (-0.61, 0.56) | 0.9323 |
| 6-month | | | | | |
| # of non-missing records | 508 | 461 | 969 | | |
| WHO5_bl, mean (std) | 13.08 (5.36) | 13.11 (5.24) | 13.10 (5.30) | 0.03 (-0.63, 0.70) | 0.92 |
| WHO5_6m, mean (std) | 13.88 (5.23) | 14.15 (5.43) | 14.01 (5.33) | 0.27 (-0.40, 0.95) | 0.4246 |
| 6-month change, mean (std) | 0.80 (4.67) | 1.03 (4.61) | 0.91 (4.64) | 0.24 (-0.35, 0.83) | 0.4228 |
| 6-month change, 95% CI | (0.39, 1.20) | (0.61, 1.46) | (0.62, 1.20) | | |
| Paired t-test P-value | 0.0001 | <.0001 | <.0001 | | |

© 2024 Agarwal AK et al. *JAMA Network Open.*

eTable 5: Leaps Comparison with Observed Data

| | Control | Intervention | Total | Difference | P Value |
|---|---|---|---|---|---|
| # of participants | 633 | 642 | 1275 | | |
| LEAPS_bl, mean (std) | 4.43 (4.00) | 4.51 (4.28) | 4.47 (4.14) | 0.08 (-0.38, 0.53) | 0.7366 |
| 6-month | | | | | |
| # of non-missing records | 517 | 473 | 990 | | |
| LEAPS_bl, mean (std) | 4.44 (4.01) | 4.43 (4.34) | 4.44 (4.17) | -0.01 (-0.53, 0.52) | 0.983 |
| LEAPS_6m, mean (std) | 5.25 (4.08) | 4.94 (4.34) | 5.10 (4.20) | -0.31 (-0.84, 0.21) | 0.2428 |
| 6-month change, mean (std) | 0.81(4.29) | 0.51 (4.64) | 0.67 (4.46) | -0.31 (-0.86, 0.25) | 0.28 |
| 6-month change, 95% CI | (0.44, 1.19) | (0.09, 0.93) | (0.39, 0.95) | | |
| Paired t-test P-value | <.0001 | 0.0178 | <.0001 | | |
| 9-month | | | | | |
| # of non-missing records | 494 | 467 | 961 | | |
| LEAPS_bl, mean (std) | 4.40 (3.94) | 4.45 (4.28) | 4.43 (4.11) | 0.05 (-0.47, 0.57) | 0.8535 |
| LEAPS_9m, mean (std) | 5.15 (4.30) | 4.17 (3.93) | 4.67 (4.15) | -0.97 (-1.50, -0.45) | 0.0003 |
| 9-month change, mean (std) | 0.74 (4.62) | -0.28 (4.12) | 0.25 (4.41) | -1.02 (-1.58, -0.47) | 0.0003 |
| 9-month change, 95% CI | ( 0.33, 1.15) | (-0.65, 0.09) | (-0.03, 0.52) | | |
| Paired t-test P-value | 0.0004 | 0.1416 | 0.0848 | | |

eTable 6: GEE with AR(1)

© 2024 Agarwal AK et al. *JAMA Network Open.*

**Correlation structure : Independent**

**GEE model on complete cases : GAD7**

| outcome | parameter | Level1 | Level2 | Estimate | LowerCL | UpperCL | P_value |
|---|---|---|---|---|---|---|---|
| GAD7 | Intercept | | | 7.6576 | 6.5991 | 8.7161 | <.0001 |
| GAD7 | arm | 1 | | 0.2471 | -0.2697 | 0.7639 | 0.3486 |
| GAD7 | arm | 0 | | 0 | 0 | 0 | . |
| GAD7 | time | 6m | | -0.2266 | -0.5897 | 0.1364 | 0.2211 |
| GAD7 | time | 9m | | -0.941 | -1.3352 | -0.5467 | <.0001 |
| GAD7 | time | bl | | 0 | 0 | 0 | . |
| GAD7 | arm*time | 1 | 6m | -0.8003 | -1.3594 | -0.2411 | 0.005 |
| GAD7 | arm*time | 1 | 9m | -0.9211 | -1.4704 | -0.3719 | 0.001 |
| GAD7 | arm*time | 1 | bl | 0 | 0 | 0 | . |
| GAD7 | arm*time | 0 | 6m | 0 | 0 | 0 | . |
| GAD7 | arm*time | 0 | 9m | 0 | 0 | 0 | . |
| GAD7 | arm*time | 0 | bl | 0 | 0 | 0 | . |
| GAD7 | age | | | -0.0674 | -0.0888 | -0.046 | <.0001 |
| GAD7 | female | | | 1.1288 | 0.4906 | 1.7671 | 0.0005 |
| GAD7 | race_cat | Asian | | -0.9581 | -1.7119 | -0.2043 | 0.0127 |
| GAD7 | race_cat | Black | | -0.3279 | -0.9004 | 0.2445 | 0.2616 |
| GAD7 | race_cat | Other | | 0.0271 | -1.2087 | 1.2628 | 0.9658 |
| GAD7 | race_cat | White | | 0 | 0 | 0 | . |
| GAD7 | hispanic | | | 0.2795 | -0.9205 | 1.4795 | 0.648 |
| GAD7 | married_or_partner | | | -0.1092 | -0.5882 | 0.3697 | 0.6549 |

**GEE model on complete cases : PHQ9**

| outcome | parameter | Level1 | Level2 | Estimate | LowerCL | UpperCL | P_value |
|---|---|---|---|---|---|---|---|
| PHQ9 | Intercept | | | 7.078 | 6.0048 | 8.1512 | <.0001 |
| PHQ9 | arm | 1 | | 0.2186 | -0.32 | 0.7573 | 0.4263 |
| PHQ9 | arm | 0 | | 0 | 0 | 0 | . |
| PHQ9 | time | 6m | | -0.3866 | -0.7685 | -0.0048 | 0.0472 |
| PHQ9 | time | 9m | | -0.8239 | -1.2286 | -0.4192 | <.0001 |
| PHQ9 | time | bl | | 0 | 0 | 0 | . |
| PHQ9 | arm*time | 1 | 6m | -0.8778 | -1.4468 | -0.3087 | 0.0025 |
| PHQ9 | arm*time | 1 | 9m | -1.042 | -1.6125 | -0.4715 | 0.0003 |
| PHQ9 | arm*time | 1 | bl | 0 | 0 | 0 | . |
| PHQ9 | arm*time | 0 | 6m | 0 | 0 | 0 | . |
| PHQ9 | arm*time | 0 | 9m | 0 | 0 | 0 | . |
| PHQ9 | arm*time | 0 | bl | 0 | 0 | 0 | . |
| PHQ9 | age | | | -0.043 | -0.065 | -0.0209 | 0.0001 |
| PHQ9 | female | | | 0.7914 | 0.1559 | 1.4268 | 0.0146 |
| PHQ9 | race_cat | Asian | | -0.8548 | -1.6263 | -0.0833 | 0.0299 |
| PHQ9 | race_cat | Black | | -0.2558 | -0.8429 | 0.3313 | 0.3931 |
| PHQ9 | race_cat | Other | | 0.3904 | -1.036 | 1.8169 | 0.5916 |
| PHQ9 | race_cat | White | | 0 | 0 | 0 | . |
| PHQ9 | hispanic | | | 0.5321 | -0.7427 | 1.8069 | 0.4133 |
| PHQ9 | married_or_partner | | | -0.5055 | -1.0005 | -0.0104 | 0.0454 |

**Correlation structure : Autoregressive**

**GEE model on complete cases : GAD7**

| outcome | parameter | Level1 | Level2 | Estimate | LowerCL | UpperCL | P_value |
|---|---|---|---|---|---|---|---|
| GAD7 | Intercept | | | 7.6385 | 6.6114 | 8.6656 | <.0001 |
| GAD7 | arm | 1 | | 0.2485 | -0.2681 | 0.7651 | 0.3457 |
| GAD7 | arm | 0 | | 0 | 0 | 0 | . |
| GAD7 | time | 6m | | -0.2347 | -0.5729 | 0.1035 | 0.1738 |
| GAD7 | time | 9m | | -0.9222 | -1.2942 | -0.5502 | <.0001 |
| GAD7 | time | bl | | 0 | 0 | 0 | . |
| GAD7 | arm*time | 1 | 6m | -0.7889 | -1.3122 | -0.2656 | 0.0031 |
| GAD7 | arm*time | 1 | 9m | -0.8723 | -1.3932 | -0.3514 | 0.001 |
| GAD7 | arm*time | 1 | bl | 0 | 0 | 0 | . |
| GAD7 | arm*time | 0 | 6m | 0 | 0 | 0 | . |
| GAD7 | arm*time | 0 | 9m | 0 | 0 | 0 | . |
| GAD7 | arm*time | 0 | bl | 0 | 0 | 0 | . |
| GAD7 | age | | | -0.0687 | -0.0897 | -0.0476 | <.0001 |
| GAD7 | female | | | 1.2174 | 0.6062 | 1.8286 | <.0001 |
| GAD7 | race_cat | Asian | | -0.8811 | -1.6056 | -0.1566 | 0.0171 |
| GAD7 | race_cat | Black | | -0.3033 | -0.8688 | 0.2621 | 0.293 |
| GAD7 | race_cat | Other | | -0.0991 | -1.2557 | 1.0575 | 0.8666 |
| GAD7 | race_cat | White | | 0 | 0 | 0 | . |
| GAD7 | hispanic | | | 0.339 | -0.7897 | 1.4678 | 0.5561 |
| GAD7 | married_or_partner | | | -0.1398 | -0.6081 | 0.3285 | 0.5585 |

**GEE model on complete cases : PHQ9**

| outcome | parameter | Level1 | Level2 | Estimate | LowerCL | UpperCL | P_value |
|---|---|---|---|---|---|---|---|
| PHQ9 | Intercept | | | 7.1471 | 6.101 | 8.1931 | <.0001 |
| PHQ9 | arm | 1 | | 0.221 | -0.3175 | 0.7594 | 0.4212 |
| PHQ9 | arm | 0 | | 0 | 0 | 0 | . |
| PHQ9 | time | 6m | | -0.3284 | -0.6878 | 0.031 | 0.0733 |
| PHQ9 | time | 9m | | -0.7522 | -1.1366 | -0.3678 | 0.0001 |
| PHQ9 | time | bl | | 0 | 0 | 0 | . |
| PHQ9 | arm*time | 1 | 6m | -0.9104 | -1.4522 | -0.3687 | 0.001 |
| PHQ9 | arm*time | 1 | 9m | -1.07 | -1.6153 | -0.5248 | 0.0001 |
| PHQ9 | arm*time | 1 | bl | 0 | 0 | 0 | . |
| PHQ9 | arm*time | 0 | 6m | 0 | 0 | 0 | . |
| PHQ9 | arm*time | 0 | 9m | 0 | 0 | 0 | . |
| PHQ9 | arm*time | 0 | bl | 0 | 0 | 0 | . |
| PHQ9 | age | | | -0.0457 | -0.0674 | -0.0241 | <.0001 |
| PHQ9 | female | | | 0.8823 | 0.2603 | 1.5043 | 0.0054 |
| PHQ9 | race_cat | Asian | | -0.6951 | -1.4813 | 0.0912 | 0.0832 |
| PHQ9 | race_cat | Black | | -0.2489 | -0.8331 | 0.3354 | 0.4039 |
| PHQ9 | race_cat | Other | | 0.1954 | -1.1532 | 1.5441 | 0.7764 |
| PHQ9 | race_cat | White | | 0 | 0 | 0 | . |
| PHQ9 | hispanic | | | 0.4325 | -0.7581 | 1.6231 | 0.4765 |
| PHQ9 | married_or_partner | | | -0.576 | -1.0642 | -0.0879 | 0.0207 |

© 2024 Agarwal AK et al. *JAMA Network Open.*
