# Supplementary material for: Digital Engagement Strategy and Health Care Worker Mental Health: A Randomized Clinical Trial
Source: JAMA Netw Open. 2024 May 24;7(5):e2410994. doi: 10.1001/jamanetworkopen.2024.10994 (PMC11127125; doi:10.1001/jamanetworkopen.2024.10994)
Supplement: Supplement 3. — Data Sharing Statement [file jamanetwopen-e2410994-s003.pdf]

Data Sharing Statement

Agarwal. Digital Engagement Strategy and Health Care Worker Mental Health. *JAMA Netw Open*. Published May 24, 2024. doi:10.1001/jamanetworkopen.2024.10994

Data
Data available: Yes
Data types: Deidentified participant data
How to access data: anish.agarwal@pennmedicine.upenn.edu
When available: With publication

Supporting Documents
Document types: Statistical/analytic code, Informed consent form
How to access documents: anish.agarwal@pennmedicine.upenn.edu
When available: With publication

Additional Information
Who can access the data: researchers whose proposed use of the data has been approved
Types of analyses: for a specified purpose
Mechanisms of data availability: with a signed data access agreement
